# Functions of Paracrine PDGF Signaling in the Proangiogenic Tumor Stroma Revealed by Pharmacological Targeting 

**DOI:** 10.1371/journal.pmed.0050019

**Published:** 2008-01-29

**Authors:** Kristian Pietras, Jessica Pahler, Gabriele Bergers, Douglas Hanahan

**Affiliations:** 1 Department of Biochemistry and Biophysics, Diabetes Center and Comprehensive Cancer Center, University of California San Francisco, San Francisco, California, United States of America; 2 Ludwig Institute for Cancer Research, Karolinska Institutet, Stockholm, Sweden; 3 Department of Neurosurgery, University of California San Franciso, San Francisco, California, United States of America; Memorial Sloan-Kettering Cancer Center, United States of America

## Abstract

**Background:**

Important support functions, including promotion of tumor growth, angiogenesis, and invasion, have been attributed to the different cell types populating the tumor stroma, i.e., endothelial cells, cancer-associated fibroblasts, pericytes, and infiltrating inflammatory cells. Fibroblasts have long been recognized inside carcinomas and are increasingly implicated as functional participants. The stroma is prominent in cervical carcinoma, and distinguishable from nonmalignant tissue, suggestive of altered (tumor-promoting) functions. We postulated that pharmacological targeting of putative stromal support functions, in particular those of cancer-associated fibroblasts, could have therapeutic utility, and sought to assess the possibility in a pre-clinical setting.

**Methods and Findings:**

We used a genetically engineered mouse model of cervical carcinogenesis to investigate platelet-derived growth factor (PDGF) receptor signaling in cancer-associated fibroblasts and pericytes. Pharmacological blockade of PDGF receptor signaling with the clinically approved kinase inhibitor imatinib slowed progression of premalignant cervical lesions in this model, and impaired the growth of preexisting invasive carcinomas. Inhibition of stromal PDGF receptors reduced proliferation and angiogenesis in cervical lesions through a mechanism involving suppression of expression of the angiogenic factor fibroblast growth factor 2 (FGF-2) and the epithelial cell growth factor FGF-7 by cancer-associated fibroblasts. Treatment with neutralizing antibodies to the PDGF receptors recapitulated these effects. A ligand trap for the FGFs impaired the angiogenic phenotype similarly to imatinib. Thus PDGF ligands expressed by cancerous epithelia evidently stimulate PDGFR-expressing stroma to up-regulate FGFs, promoting angiogenesis and epithelial proliferation, elements of a multicellular signaling network that elicits functional capabilities in the tumor microenvironment.

**Conclusions:**

This study illustrates the therapeutic benefits in a mouse model of human cervical cancer of mechanism-based targeting of the stroma, in particular cancer-associated fibroblasts. Drugs aimed at stromal fibroblast signals and effector functions may prove complementary to conventional treatments targeting the overt cancer cells for a range of solid tumors, possibly including cervical carcinoma, the second most common lethal malignancy in women worldwide, for which management remains poor.

## Abstract 2

Douglas Hanahan and colleagues investigate a paracrine regulatory circuit centered upon PDGF receptor signaling in cancer-associated fibroblasts and pericytes of a mouse model of cervical carcinogenesis.

## Editors' Summary

**Background.:**

Cancers—disorganized, life-threatening masses of cells—develop when cells acquire genetic changes that allow them to divide uncontrollably and to move into (invade) other tissues. Interactions with ostensibly normal cells in the tissue surrounding the tumor (the stroma) support the growth of these abnormal cells. The stroma contains endothelial cells and pericytes (which line the inside and coat the outside, respectively, of blood vessels), cancer-associated fibroblasts, and some immune system cells. Together, these cells support angiogenesis (the formation of a blood supply, which feeds the tumor), produce factors that stimulate tumor cell growth, and facilitate tumor cell invasion into surrounding tissues. One type of tumor with a prominent stromal compartment is cervical cancer. Precancerous changes in the epithelial cells lining the cervix (the structure that connects the womb to the vagina) are usually triggered by infection with human papillomavirus. Some of these early lesions, which are known as cervical intraepithelial neoplasias (CINs), develop into invasive cervical cancer, which is treated by surgery followed by chemotherapy or radiotherapy.

**Why Was This Study Done?:**

The outlook for women whose cervical cancer is detected early is good but only 15%–30% of women whose cancer has spread out of the cervix survive for five years. If, as researchers believe, the stromal compartment is important in the development and growth (neoplastic progression) of cervical cancer, it might be possible to help these women by specifically targeting the cells in the stroma. However, relatively little is known about the role that the stroma plays in the neoplastic progression of cervical cancer or how it is regulated other than that a protein called platelet-derived growth factor (PDGF), which is made by the tumor cells, might be involved in its formation. In this study, the researchers have used a mouse model of cervical cancer (HPV/E_2_ mice) to investigate PDGF signaling in the tumor stroma. HPV/E_2_ mice develop CINs before they are three months old; by five months of age, 90% of them have invasive cervical cancer.

**What Did the Researchers Do and Find?:**

The researchers report that PDGF was expressed in the cervixes of normal and HPV/E_2_ mice, mainly by epithelial cells, and that PDGF receptors (cell-surface proteins that bind PDGF and send a message into the cell that alters the expression of other proteins) were expressed on cells within normal stroma and in fibroblasts and pericytes in the stroma surrounding CINs and tumors (but not on the cancer cells). The expression of PDGF and its receptors increased slightly during tumor progression. Treatment of the HPV/E_2_ mice with imatinib, an inhibitor of PDGF signaling, slowed the progression of precancerous lesions, impaired the growth of invasive cancers, and reduced the number of blood vessels formed in the tumors and the coverage of these vessels with pericytes. Other experiments indicate that imatinib had these effects because its inhibition of stromal PDGF receptors suppressed the expression of FGF-7 (a factor that encourages epithelial cell division) and FGF-2 (a proangiogenic factor) by cancer-associated fibroblasts. Finally, as in HPV/E_2_ mice, FGF-2 and PDGF receptors were expressed in the stroma of human cervical cancers whereas PDGF was expressed in the cancer cells.

**What Do These Findings Mean?:**

These findings suggest that PDGF receptor signaling in the stromal cells associated with cervical tumors in mice has a functional role during tumor progression. More specifically, they suggest that PDGF released by the tumor cells triggers PDGF signaling in the stromal cells, which increases the expression of factors that both directly and indirectly stimulate the growth of the tumor cells. Confirmation of this scheme will require additional experiments in mouse models of cervical cancer and the careful examination of more human material. Importantly, although approaches that work in mice do not always work in people, the current findings suggest that targeted therapeutics that prevent the stromal support of tumor growth (such as inhibitors of PDGF receptor signaling) might provide a complementary approach to conventional treatments that target the cancer cells themselves.

**Additional Information.:**

Please access these Web sites via the online version of this summary at http://dx.doi.org/10.1371/journal.pmed.0050019.

- The US National Cancer Institute provides information on all aspects of cancer, including information about cervical cancer (in English and Spanish)
- The UK charity Cancerbackup also provides information on all aspects of cancer, including information on cervical cancer and on imatinib
- Wikipedia has pages on platelet-derived growth factor, on PDGF receptors, and on imatinib (note that Wikipedia is a free online encyclopedia that anyone can edit; available in several languages)

## Introduction

It is increasingly accepted that cancer results from the concerted performance of genetically altered tumor cells interacting with ostensibly normal cell types that together constitute the tumor microenvironment [1]. In addition to the endothelial cells forming the tumor vasculature, attention is now focused on other elements of the stromal compartment, i.e. carcinoma-associated fibroblasts (CAFs), vascular pericytes, and infiltrating inflammatory cells. These cell types are being implicated as functionally important for tumorigenesis, by providing proliferative and antiapoptotic regulatory factors, supporting tumor angiogenesis, and facilitating invasion [2–7]. Recent studies have described extensive changes in the expression profile of cells within the stroma compared to their normal counterparts [8–11]. Among the factors implicated in the development of a reactive stroma, in particular the recruitment and phenotypic character of CAFs, are members of the transforming growth factor-β and the platelet-derived growth factor (PDGF) families [4,6,12]. The involvement of different PDGF isoforms in both autocrine and paracrine stimulation of tumor growth has been extensively studied [13,14]. The appreciation that PDGFs serve to regulate the reactive stromal phenotype of tumors has come from studies demonstrating that expression of PDGF promotes the establishment of a well-vascularized and prominent stroma in transplant models of melanoma [15], breast carcinoma [16], squamous carcinoma [17], fibrosarcoma [18], and lung carcinoma [19], consequently enhancing tumor growth.

Cervical carcinoma is the second most common malignant disease among women worldwide and the most common cause of cancer death in many less-developed countries [20]. The primary etiologic agents for cervical cancer are human papilloma viruses (HPV). In particular, invasive cervical and anogenital tumors are epidemiologically associated with chronic infection by HPV type 16 (HPV16) and related “high risk” viral subtypes; the viral genomes contain two transcription units, E6 and E7, encoding proteins that bind to and inactivate the p53 and pRb tumor suppressors, respectively, facilitating unchecked cell cycle progression and genomic instability [21]. Even though cervical cancers are successfully managed by surgery and chemoradiotherapy if detected at an early stage, the management of late-stage disease remains poor [20].

Directed expression of the oncogenes contained in the early region of HPV16 to their apparent target cell type in humans, the basal squamous epithelial cell, by way of expression under control of the keratin 14 (K14) promoter in transgenic mice, leads to the formation of squamous cell carcinomas (SCC) of the skin; the skin tumors arise with 50% penetrance between 6–12 mo of age in the FVB/n mouse strain [22]. If the normally cyclic estrogen levels (17β-estradiol [E_2_]) in young female transgenic mice are maintained by implantation of slow-release estrogen pellets (HPV/E_2_ mice), invasive squamous carcinomas of the cervix develop via transition through distinctive premalignant stages, i.e. cervical intraepithelial neoplasias (CIN) [23]. Following relatively synchronous progression through CIN1 to CIN2 and CIN3 lesions, cervical carcinomas begin to appear at 3.5 mo of age; 6 wk later, approximately 90% of the HPV/E_2_ mice present with invasive cervical cancer [24], well before skin cancers begin to appear. The HPV16/E_2_ mouse model of cervical carcinoma closely resembles the human counterpart with respect to the progressively intense angiogenic phenotype, with increased bioavailability of vascular endothelial growth factor (VEGF) as one feature [24,25]. Studies from our laboratory have also demonstrated the importance of matrix metalloproteinase-9 (MMP-9), primarily supplied by infiltrating macrophages, in the activation of the angiogenic switch in this model [24], consistent with the presence of infiltrating macrophages and MMP-9–expressing cells in human cervical neoplasias and carcinomas [26].

The stromal compartment is prominent in cervical carcinoma, and recent studies have identified numerous changes in the gene expression pattern of stromal cells in malignant cervical tissue compared to nonmalignant tissue [9,27], suggestive of altered functions. An evident question involves the importance of the stromal compartment for neoplastic progression, and if important, its roles and regulation. We sought to address the hypothesis that stromal support functions in cervical carcinogenesis are a) functionally important and b) a potential therapeutic target. To do so we used a genetically engineered mouse model of cervical cancer to assess the impact of mechanism-based pharmacological targeting of stromal support functions, in particular those of cancer-associated fibroblasts.

## Materials and Methods

### Animal Care and Generation of HPV/E_2_ Mice

All animal experimentation described herein was approved by the local Committee for Animal Research. HPV/E_2_ mice were generated as described previously [22,23,28]. Briefly, at 1 mo of age, virgin, female, heterozygous transgenic *K14-HPV16* mice or nontransgenic FVB/n mice were anesthetized and slow-release E_2_ pellets (0.05 mg over 60 d; Innovative Research of America) were implanted subcutaneously in the dorsal back. Additional pellets were implanted at 3 and 5 mo of age. Mice were monitored throughout the experiments for complications due to the dysplastic nature of their skin or to the E_2_ treatment.

### Tissue Preparation and Histology

Mice were anesthetized with 2.5% Avertin and heart perfused with PBS followed by ice-cold 10% zinc-buffered formalin. Subsequently, the vagina, cervix, and uterine horns were excised and either post-fixed for 1 h at 4 °C followed by embedding in paraffin or immediately frozen in OTC. The entire tissue was serially sectioned (10-μm sections) and every tenth slide was subjected to hematoxylin and eosin staining for grading in a blinded fashion, as described [29]. Tumor volume was determined using the formula *V* = 2/3 × *A* × *Z*, where *A* is the largest cross-sectional area, as determined by imaging using a Zeiss Axioskop 2 plus microscope and OpenLab software (Improvision), and *Z* is the depth of the tumor as determined through the serial sections.

### Treatment of Mice and Preparation of Adenoviral FGF-Trap

Imatinib (Gleevec; Novartis Pharma) was purchased from the University of California San Francisco (UCSF) Medical Center pharmacy. Imatinib was delivered by oral gavage in 100-μl PBS at a dose of 150 mg × kg^−1^ × day^−1^, divided in a morning dose of 50 mg × kg^−1^ × day^−1^ and an afternoon dose of 100 mg × kg^−1^ × day^−1^. Mice were treated daily either from 3.5–5 mo of age or from 5–6 mo, and closely monitored for drug-induced side effects. Three hours before they were humanely killed, mice were injected intraperitoneally with 10 μl/g of a 10 mM bromo-deoxyuridine (BrdU) solution. Adenovirus FGF-trap was developed and described previously [30,31], and preparation of high-titer adenovirus was subcontracted to Vector Biolabs. The adenovirus was delivered to 3.5-mo-old HPV/E_2_ mice via tail vein injection at 0.3–1 × 10^9^ viral particles/mouse. The mice were humanely killed 14 d later, and the cervixes were prepared for analysis as described above. Functional-grade rat monoclonal anti-PDGFR-α (APA5) was purchased from eBiosciences, and rat monoclonal anti-PDGFR-β (APB5 [32]) was produced at high purity from a hybridoma. The 3.5-mo-old mice were treated with 0.5 mg/mouse of anti-PDGFR-α and 0.5 mg/mouse anti-PDGFR-β injected intraperitoneally daily for 3 d. Control mice were given 1 mg/mouse and day of functional-grade rat IgG (Jackson ImmunoResearch). Mice were humanely killed 24 h after the last injection, then tissues collected and flash frozen in liquid nitrogen for subsequent RT-PCR analysis.

### Immunostaining

Frozen sections were air dried and fixed in ice-cold acetone for 10 min. Paraffin-embedded sections were de-paraffinized, endogenous peroxidase activity was quenched with 0.3% H_2_O_2_ for 30 min at room temperature (rt). Following stabilization in PBS for 2 × 5 min, sections were blocked using a 1:1 mix (blocking solution) of serum-free protein block (DAKO) and 0.5% blocking reagent in PBS (NEN Life Science Products) for 1–2 h at rt. After aspiration, antibodies diluted in blocking solution were applied and incubated at 4 °C overnight. Antibodies and dilutions used were: PDGFR-α (1:100, APA5; eBiosciences), PDGFR-β (1:100, APB5; eBiosciences), NG2 (1:800, AB5320; Chemicon), PDGF-CC (6 μg/ml [33]), CD31 (1:100, MEC 13.3; Pharmingen), activated caspase-3 (1:100, Asp175; Cell Signaling Technology), BrdU (1:10, BrdU labeling and detection kit; Roche), FGF-2 (1:100, sc-79-G; Santa Cruz Biotechnology). Slides were washed 3 × 10 min in PBS + 1% bovine serum albumin (BSA; washing solution) and subsequently incubated 2 h at rt with appropriate conjugated secondary antibodies (fluorescently labeled, 1:100; Jackson Immunolaboratories; and HRP-conjugated, 1:100; Vector laboratories) diluted in blocking solution. The signal from HRP-conjugated secondary antibodies was amplified using the Vectastain ABC system according to the manufacturer's instructions (Vector laboratories). Following 3 × 10 min wash in washing solution, sections were mounted for microscopic evaluation. The specificity of all stainings was confirmed by omitting the primary antibody. Moreover, for immunohistochemical staining of FGF-2, the specificity of the primary antibody was determined by preincubating the antibody with a 4-fold molar excess of human FGF-2 for 1 h at rt prior to the procedure described.

### Quantification of Vessel Density, Pericyte Coverage, Apopotic Index, and Proliferative Index

All measurements were performed using the OpenLab software (Improvision) on at least ten different sections from five different mice of similar histological stage in each treatment group. Vessel density was calculated as percentage of the stromal cross-sectional surface area that stained positively for CD31, or as number of CD31-positive vessel structures per high power field (400× magnification). Pericyte coverage was calculated as percentage of CD31-positive vessels that displayed any associated NG2-positive cells. The apoptotic and proliferative indexes were measured as percentage of epithelial cells staining positively for activated caspase-3 or BrdU, respectively.

### RNA Extraction and Quantitative RT-PCR Analysis

The cervixes from at least five mice of the same age and treatment were used to make up a pool of tissue for the analyses. The mice that were used to make up the various pools were N/E_2_, 5-mo-old nontransgenic, estrogen-treated FVB/n mice; CIN3, 3-mo-old HPV/E_2_ mice; SCC, 5-mo-old HPV/E_2_ mice; sham-treated mice, 4-mo-old HPV/E_2_ mice; imatinib-treated mice, 4-mo-old HPV/E_2_ mice that were treated as described above from 3.5–4 mo of age. The cervix was excised, snap frozen in liquid N_2_, and ground to a powder using a mortar and pestle. Following further homogenization using an electrical homogenizer, total RNA was extracted using the RNeasy mini-kit (Qiagen) under RNase-free conditions. After quantification of yield, 1-μg total RNA was reverse transcribed into cDNA by incubation at 65 °C for 5 min with random primers (Invitrogen), followed by incubation at 48 °C for 60 min in a mix of first-strand incubation buffer (Invitrogen), 10 mM dNTPs (RNase-free; Roche), and Superscript III reverse transcriptase (Invitrogen) in a total volume of 40 μl. The yield of total RNA input to cDNA output was assumed to be 1:1. Taqman analyses of expression levels were subsequently performed in triplicate for each sample by the UCSF Genome Analysis core facility using assays from Applied Biosystems (PDGF-A, Mm00833533_m1; PDGF-B, Mm00440678_m1; PDGF-C, Mm00480205_m1; PDGF-D, Mm00546829_m1; PDGFR-α, Mm00440701_m1; PDGFR-β, Mm00435546_m1; FGF-1, Mm00438906_m1; FGF-2, Mm00433287_m1; FGF-7, Mm00433291_m1; c-kit, Mm00445212_m1; SCF, Mm00442972_m1; VEGF, Mm00437304_m1; angiopoietin-1, Mm00456503_m1; angiopoietin-2, Mm00545822_m1; ephrin A1, Mm00438660_m1; ephrin B2, Mm00438670_m1; EGF, Mm00438696_m1; TGF-α, Mm00446231_m1; IGF-1, Mm00446231_m1; IGF-2, Mm00439563_m1; and HGF, Mm00690363_m1). Alternatively, analysis of expression of FGF-2 and L19 was performed using a Rotor Gene RG-3000A (Corbett Research) with SYBR green mix (Invitrogen) and the following primers: L19 5′-GGT GAC CTG GAT GAG AAG GA-3′ *(forward);* 5′-TTC AGC TTG TGG ATG ATG TGC TC-3′ *(reverse);* FGF-2 5′-GGC TGC TGG CTT CTA AGT GT-3′ *(forward);* 5′-CCG TTT TGG ATC CGA GTT TA-3′ *(reverse).*

Expression levels are expressed as percentage of the expression level for the housekeeping gene *L19*, but similar results were obtained comparing to the housekeeping gene *mGUS*.

### Immunoprecipitation and Western Blotting

Tissue lysates from the cervixes of 4-mo-old HPV/E_2_ mice treated or not with imatinib for 2 wk were prepared in a RIPA buffer (150 mM NaCl, 1% Triton X-100, 0.5% NaDeoxycholate, 0.1% SDS, 50 mM Tris-Hcl [pH 8.0]). Immunoprecipitation was performed using a pool of antibodies against FGF-2 (AB5396, Chemicon; AB-07, Advanced Targeting Systems; and sc-79-G, Santa Cruz Biotechnology). After collection of immune complexes using Protein A sepharose beads (GE Healthcare) and SDS-PAGE, western blot analysis was performed using a 2 μg/ml solution of the sc-79-G antibody (Santa Cruz Biotechnology).

### Flow Cytometry and Qualitative RT-PCR

Six 3.5-mo-old HPV/E_2_ mice were humanely killed and the cervixes excised and cut into small pieces (1 × 1 mm). The tissue was subsequently submerged in PBS containing 1% BSA and 5% cell dissociation buffer (Gibco) (FACS buffer), supplemented with 0.25% (w/v) type I collagenase (Worthington), 0.25% (w/v) type II collagenase (Worthington), and 0.05% (w/v) DNAse (Sigma). Digestion was carried out at 37 °C for 18 min with constant stirring and occasional mechanical disruption using a plastic pipette. FACS buffer containing 10% fetal bovine serum (FBS; Gibco) was added, and the solution was filtered through a 70-μm cell strainer. After centrifugation and aspiration of the buffer, cells were resuspended in ACK buffer (Cambrex) for 5 min at rt to lyse red blood cells. Cells were washed once in FACS buffer containing 10% FBS and diluted to a concentration of 10^6^ cells/ml. Subsequently, F_c_ receptors were blocked using a mix of antibodies towards CD16/CD32 (eBiosciences) for 10 min on ice. Antibodies for labeling of cells were added for 15 min on ice at 1:50 dilution, and consisted of PE-labeled anti–c-kit (ack-45; BD-Pharmingen) and biotinylated anti-PDGFR-α and -β (APA5, APB5; eBiosciences), followed by FITC-labeled streptavidin (BD-Pharmingen). After two washes in FACS buffer, and addition of 1 μg/ml propidium iodide, viable cells were sorted using a fluorescence activated cell sorting (FACS) machine. Cells were collected in 350 μl of RLT buffer from Qiagen's RNeasy mini kit, and total RNA was purified and subsequently amplified and transcribed into cDNA using the Ovation amplification system (Nugene) according to the manufacturer's instruction. PCR analysis (*T*_m_ = 58 °C) was performed using the following primers: K14 5′-TTC CGG ACC AAG TTT GAG AC-3′ *(forward);* 5′-CCT CGT GGT TCT TCT TCA GG-3′ *(reverse);* vimentin 5′-GCA CTA ACG AGT CCC TGG AG-3′ *(forward);* 5′-TCC AGC AGC TTC CTG TAG GT-3′ *(reverse);* FGF-2 see above.

### Statistical Analysis

All measurements are depicted as average ± standard error of the mean. Statistical analysis of tumor volume was performed using a two-tailed Mann-Whitney *U* test. Statistical analysis of tumor incidence was performed using a χ^2^ test. Statistical analysis of gene expression and tumor characteristics, such as vessel density, pericyte coverage, apoptotic and proliferative index, etc. was performed using a two-tailed, unpaired Student *t*-test. A *p*-value below 0.05 was considered statistically significant.

## Results

### PDGF Ligands and Receptors Are Up-Regulated during Cervical Carcinogenesis in HPV/E_2_ Mice

**Figure 1:**
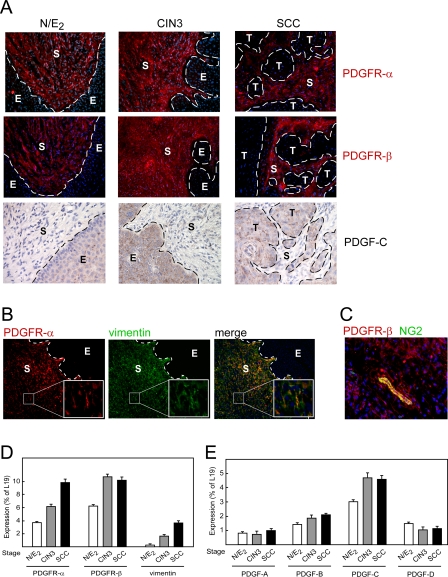
Expression of the PDGF Receptors during Cervical Carcinogenesis The cervixes of estrogen-treated normal mice (N/E_2_), HPV/E_2_ mice with CIN3 lesions (at 3 mo of age), or with cervical squamous cell carcinomas, SCC (at 5 mo of age) were compared. Expression patterns were analyzed in at least five different mice of the same genotype or similar histological stage. Dotted line marks the epithelium-stroma boundary. E, epithelium; S, stroma; T, tumor. (A) Immunostaining of PDGFR-α and -β (200× magnification; PDGFR, red; cell nuclei/DAPI, blue). Immunostaining of PDGF-CC (400× magnification). (B) Coexpression of PDGF receptors and the mesenchymal cell marker vimentin in stromal fibroblasts of the transformation zone of HPV/E_2_ mice (200× magnification; PDGF-α receptors, red; vimentin, green; merge; cell nuclei/DAPI, blue). (C) Coexpression of PDGFR-β and the pericyte marker NG2 in the cervical stroma of the transformation zone of HPV/E_2_ mice (400× magnification; PDGFR-β, red; NG2, green; cell nuclei/DAPI, blue). (D and E) Quantitative PCR analysis of the expression of PDGF receptors (D) and ligands (E) in the cervixes of estrogen-treated normal or HPV mice with progressing tumor development. Error bars indicate the standard error of the mean.

The transformation zone between squamous and columnar epithelium of the uterine cervix is implicated as the site of origin of the human cancer [34]. Similarly, in *K14-HPV16* female transgenic mice whose estrogen levels are maintained by time-release implants (HPV/E_2_ mice), incipient neoplasias first appear in the transformation zone, arising out of the HPV-16 oncogene-expressing squamous epithelium; the progressive neoplastic lesions are associated with an aberrant (“reactive”) stroma [28]. Motivated by the well-established association of PDGF signaling with regulation of fibroblast phenotypes and by our previous observations that vascular pericytes in tumors are dependent on PDGF signaling [35–37], we investigated the expression of PDGF ligands and receptors during cancer progression in HPV/E_2_ mice. In normal, estrogen-treated female mice (N/E_2_ mice), as well as in HPV/E_2_ mice, both PDGF receptor-α and -β were expressed by cells populating the stroma of the cervical transformation zone, as revealed by immunostaining (Figure 1A). The expression of PDGF receptors persisted in the apparently denser stroma of CIN3 and SCC lesions (Figure 1A). Immunostaining revealed that stromal cells expressing both PDGF α- and β-receptors coexpress the mesenchymal cell marker vimentin, demonstrating together with morphological (spindle-shaped cells with indented nuclei) and histological (patterns of tissue localization ) criteria that these cells are fibroblasts (Figure 1B and unpublished data). Additionally, PDGF β-receptor was also expressed by pericytes, as indicated by costaining with the mural cell marker NG2 (Figure 1C). Quantitative PCR analysis revealed that the cervical expression of both PDGF α- and β-receptors was increased concomitant with neoplastic progression; the cervix of HPV/E_2_ mice with SCC displayed a 2.7-fold and 1.6-fold increase in expression of PDGF α- and β-receptor, respectively, compared to N/E_2_ mice (Figure 1D). Expression of vimentin was found to be similarly increased (Figure 1D), indicating that the elevated expression of PDGF receptors in the tissue largely results from an increased cellularity of the stroma.

All four PDGF ligand genes were found to be expressed at readily detectable levels in the cervixes of control N/E_2_ mice (Figure 1E). Gene expression of the most abundant PDGF ligand, PDGF-C, was modestly increased during the course of tumorigenesis in HPV/E_2_ mice (Figure 1E). Immunohistochemical analysis confirmed the expression of PDGF-CC, and revealed that the primary source of PDGF-CC was the epithelium, both in control N/E_2_ mice and in HPV/E_2_ mice (Figure 1A). Due to the small mass of the mouse cervix and a lack of reagents with high sensitivity and specificity, we were unable to quantitate the level of PDGF-CC protein during the course of cervical tumor progression.

### A PDGF Receptor Kinase Inhibitor Delays Progression and Slows Growth of Cervical Carcinomas

**Figure 2:**
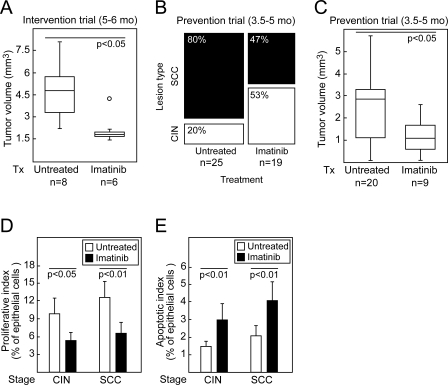
Effects of Treating HPV/E_2_ Mice with the PDGF Receptor Inhibitor Imatinib (A) Tumor volume of carcinomas in the uterine cervixes of cohorts of mice treated (or not) with imatinib for 4 wk in an intervention trial, ending at 6 mo of age. Mann-Whitney *U* test, *U* = 5, *p* < 0.05. (B and C) Incidence (χ^2^ test, χ^2^ = 5.1, *p* < 0.05) (B) and tumor volume (Mann-Whitney *U* test, *U* = 46, *p* < 0.05) (C) of invasive tumors in the uterine cervix following a 6-wk-long prevention trial ending at 5 mo of age. Mice without carcinomas had CIN2–3 lesions. (D and E) Analysis of the proliferative (Student *t*-test, t(CIN3) = 2.3, *p* < 0.05; t(SCC) = 3.6, *p* < 0.01) (D) and apoptotic index (Student *t*-test, t(CIN3) = 3.1, *p* < 0.01; *t*(SCC) = 3.6, *p* < 0.01) (E) of CIN and SCC lesions in the cervixes of HPV/E_2_ mice treated in the prevention trial with vehicle or imatinib. Error bars indicate the standard error of the mean.

Having documented the expression of PDGF ligands in the neoplastic epithelia and PDGF receptors in the stroma at all stages in cervical carcinogenesis, we sought to assess the functional significance and therapeutic potential of PDGF signaling by pharmacological inhibition at distinct stages of cervical carcinogenesis. We chose to disrupt PDGF receptor signaling with the kinase inhibitor imatinib (Gleevec) [38], which we and others have documented to be effective at inhibiting PDGF receptor in mice [39,40]. To establish the efficacy of imatinib treatment in the cervix, we immunoprecipitated PDGF receptor-α from cervical tissue lysates from HPV/E_2_ mice treated twice daily for 2 wk with imatinib. Western blotting for activated PDGF receptor-α revealed that the phosphotyrosine content of the receptor was reduced by 72% following treatment with imatinib (Figure S1). The first therapeutic trial was initiated at the age of 5 mo, when more than 80% of the mice display overt carcinoma lesions in the cervix [24]. The trial continued for 1 mo (an Intervention Trial). The median tumor volume at this temporally defined endpoint was decreased by 61% following imatinib treatment, demonstrating that this agent can impair the maintenance and growth of preexisting cervical tumors (Figure 2A; Mann-Whitney *U* test, *U* = 5, *p* < 0.05).

We next conducted an earlier stage Prevention Trial aimed at premalignant disease. Treatment of the HPV/E_2_ mice with imatinib was initiated at 3.5 mo of age, at which time more than 90% of the mice harbor CIN3 lesions [24]; the inhibition was continued for 6 wk. The incidence of cervical carcinoma at the defined endpoint in sham-treated HPV/E_2_ mice was 80%, in close agreement with earlier studies (Figure 2B, untreated cohort; and [24]). Following treatment with imatinib, the incidence of cervical carcinomas was significantly reduced, to 47% (Figure 2B; χ^2^ test, χ^2^ = 5.1, *p* < 0.05). Moreover, imatinib reduced the median volume of tumors that did form by 61% (Figure 2C; Mann-Whitney *U* test, *U* = 46, *p* < 0.05).

In accordance with the impaired tumor growth in both the Intervention and the Prevention Trial settings, treatment with imatinib significantly lowered the cell proliferation index (Figure 2D; Student *t*-test, *t*(CIN3) = 2.3, *p* < 0.05; *t*(SCC) = 3.6, *p* < 0.01) and increased the apoptotic index (Figure 2E; Student *t*-test, *t*(CIN3) = 3.1, *p* < 0.01; *t*(SCC) = 3.6, *p* < 0.01) of the transformed squamous epithelial cells in both premalignant CIN3 and invasive cervical carcinomas. Since the PDGF receptors were not expressed in the cancer cells, we sought other explanations for these effects on proliferation, apoptosis, and neoplastic progression.

### Another Imatinib Target, c-kit, Is Not Up-Regulated during Cervical Carcinogenesis

The inhibitory profile of imatinib (with high affinity for the PDGF α- and β-receptors, c-kit, and abl) raised an additional possibility, that its activity against c-kit or abl could be a factor in the observed antiproliferative effects. Indeed, a putative autocrine loop in squamous epithelial cervical cancer cells involving c-kit and its ligand (stem cell factor [SCF]) has been described for a small subset of human cervical cancers [41]. We therefore analyzed c-kit and SCF expression. Both were found to be expressed at relatively low levels, and neither was up-regulated in the stages of neoplastic progression in HPV/E_2_ cervix; indeed, c-kit levels declined despite the substantial expansion in the (neoplastic) epithelial compartment in CIN3 and cervical carcinomas (Figure S2A). Immunostaining revealed that c-kit was expressed exclusively by mast cells in the stromal compartment and at exceedingly low levels in a subset of squamous epithelial cells in the cell layers immediately above the basal keratinocytes (Figure S2B). Taken together, we consider it unlikely that imatinib is acting directly on the neoplastic basal epithelial cells to produce the observed growth inhibition. Additional studies with more selective inhibitors of c-kit or abl will be required to definitively confirm this conclusion. This consideration notwithstanding, the functional studies described below support the inference that imatinib's inhibition of PDGF receptors on CAFs forms the predominant basis for its perturbations of cervical carcinogenesis and the resultant conclusion that PDGF signaling is instrumental.

### Imatinib Reduces Vascular Density and Pericyte Coverage of Cervical Carcinomas

**Figure 3:**
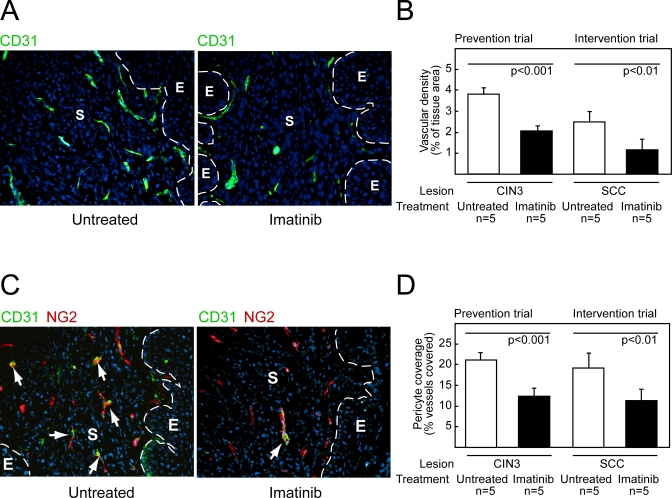
The Angiogenic Phenotype Is Impaired by Imatinib Therapy (A) Representative immunostaining of blood vessels (CD31, green) in the cervical transformation zone of HPV/E_2_ mice treated with vehicle or imatinib. Vessel density was analyzed in at least ten sections from five different mice of similar histological stage. Magnification is 200×; cell nuclei/DAPI, blue; dotted line marks the epithelium-stroma boundary. E, epithelium; S, stroma. (B) Quantification of vessel density in the cervical transformation zone of HPV/E_2_ mice treated in a prevention trial from 3.5 mo to 5 mo of age or in an intervention trial from 5 mo to 6 mo with vehicle or imatinib. Student *t*-test, *t*(CIN3) = 4.6, *p* < 0.001, *t*(SCC) = 3.8, *p* < 0.01. (C) Visualization of pericytes and endothelial cells, using the markers NG2 (green) and CD31 (red), respectively. Pericyte coverage was analyzed in at least ten sections from five different mice of similar histological stage. Magnification is 200×; cell nuclei/DAPI, blue; dotted line marks the epithelium-stroma boundary; arrows indicate coexpression. E, epithelium; S, stroma. (D) Quantification of pericyte coverage in the cervical transformation zone of HPV/E_2_ mice following a Prevention Trial or an Intervention Trial. Student *t*-test, *t*(CIN3) = 4.5, *p* < 0.001, *t*(SCC) = 3.6, *p* < 0.01. Error bars indicate the standard error of the mean.

Since previous studies had shown that imatinib and other PDGF receptor inhibitors destabilized the tumor vasculature in different tumor types, we sought to assess the effects of imatinib-mediated interference of PDGF receptor signaling on the angiogenic vasculature. The vasculature in CIN 2/3 lesions and cervical carcinomas in the HPV/E_2_ mice shows signatures of chronic angiogenesis similar to those evident in the cognate human lesions, including an increased density of dilated and tortuous microvessels proximal to the hyperproliferative epithelium. As seen in Figure 3A and 3B, imatinib caused a reduction in lesional neovascularization; the blood vessel density was diminished by 45% and 52% in CIN3 lesions and SCC, respectively, following treatment with imatinib (Figure 3B; Student *t*-test, *t*(CIN3) = 4.6, *p* < 0.001, *t*(SCC) = 3.8, *p* < 0.01). Since PDGF receptors are expressed by pericytes in the cervixes of HPV/E_2_ mice (Figure 1C), and since inhibition of PDGF receptor signaling by imatinib and other drugs is known to dissociate pericytes from blood vessels in tumors [35–37,42], we also analyzed the pericyte coverage of capillaries. Immunohistochemical staining for the pericyte marker NG2 revealed that blood vessels within the transformation zone of the normal cervix are associated with pericytes to a comparatively low degree; only 21% of blood vessels displayed any attached pericytes (Figure 3C and 3D). This observation was confirmed using desmin as an alternative pericyte marker (unpublished data). In accordance with results from other cancer models [35–37,42], imatinib reduced the fraction of capillaries associated with pericytes in cervical premalignant and SCC lesions of HPV/E_2_ mice by 42% and 39%, respectively (Student *t*-test, *t*(CIN3) = 4.5, *p* < 0.001, *t*(SCC) = 3.6, *p* < 0.01). Thus imatinib affected the angiogenic vasculature in two ways: it was antiangiogenic, reducing the number of vessels, and it reduced the coverage of those vessels by pericytes.

Activated macrophages have been reported to express PDGF receptors [43]. In previous studies, we have implicated macrophage-supplied MMP-9 as a factor in the angiogenic switch in premalignant lesions in the cervixes of HPV/E_2_ mice [24]. Therefore, we investigated whether the cervixes from HPV/E_2_ mice treated with imatinib displayed a reduction of MMP-9–expressing cells. Immunohistochemical analysis revealed no difference in the number of cells expressing MMP-9 or in the number of macrophages in the cervixes from imatinib-treated mice (Figure S3A and S3B). Additionally, no change in the abundance of other constituent cell types of the neoplastic cervix, such as leukocytes, mast cells, NK cells, or dendritic cells, were observed following treatment with imatinib (Figure S3B).

### A Screen for Molecular Effectors of Imatinib's Effects Revealed FGF-7 and FGF-2

**Figure 4:**
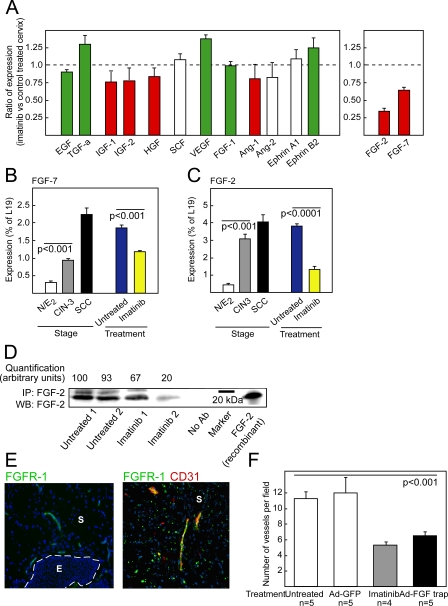
Expression of FGF-2 Is Repressed by Imatinib in Preclinical Trials, and Treatment with FGF-Trap Impairs Angiogenesis in the Neoplastic Cervix (A) Quantitative RT-PCR analysis evaluating expression of a set of growth and/or angiogenic regulatory factors in the neoplastic cervixes of 4-mo-old HPV/E_2_ mice treated with imatinib for 2 wk. The results are expressed as the ratio of expression (as percentage of the ribosomal protein gene *L19*) of imatinib-treated mice versus vehicle-treated mice. Bar colors indicate relative expression levels during the neoplastic progression (green = down-regulated expression compared with normal estrogen-treated cervix; red = up-regulated expression compared with normal estrogen-treated cervix; and white = unchanged expression compared with normal estrogen-treated cervix). (B and C) Quantitative RT-PCR analysis of FGF-7 (Student *t*-test, *t* = 15.9, *p* < 0.001) (B) and FGF-2 (Student *t*-test, *t* = 13.7, *p* < 0.001) (C) expression in the cervixes of: estrogen-treated normal mice (N/E_2_); HPV/E_2_ mice with CIN3 lesions (3 mo) or SCC (5 mo) and HPV/E_2_ mice treated from 3.5 mo to 4 mo of age with vehicle or imatinib. (D) Western blot (WB) analysis of FGF-2 expression following immunoprecipitation (IP) of FGF-2 from tissue lysates of neoplastic cervixes of mice untreated or treated with imatinib for 2 wk. Two individual tissue lysates are shown for each treatment, and every lysate for each treatment group consisted of the combined cervixes from five mice. Omission of the immunoprecipitating antibody was used as a negative control (No Ab-lane), and 50 ng of recombinant mouse FGF-2 was used as a positive control. Densitometric quantification is shown normalized to lane 1. (E) Immunostaining for the mitogenic signaling receptor for FGF-2, i.e., FGF receptor-1 (FGFR-1), in CIN3 lesions of the uterine cervix from HPV/E_2_ mice. Expression of FGFR-1 (green) was predominantly detected in the stroma and colocalized with a marker for endothelial cells (red, CD31). The expression pattern was analyzed in at least five different mice of similar histological stage. Parameters: 200× magnification; cell nuclei/DAPI, blue; dotted line marks epithelium–stroma boundary. Similar results were seen in analysis of cervical carcinoma lesions (unpublished data). Note that the scattered punctate shapes distal from the vasculature are non–cell-associated debris derived from the secondary antibody, as revealed by evaluation at high magnification and analysis of tissue sections in which the primary antibody was omitted. E, epithelium; S, stroma. (F) Quantification of vessel density in the cervical transformation zone of HPV/E_2_ mice at 4 mo of age following a 2-wk treatment with imatinib or 2 wk after a single treatment with adenoviral delivery of FGF-trap or control GFP. Student *t*-test, *t* = 5.8, *p* < 0.001. Error bars indicate the standard error of the mean.

The evidently paracrine impairment, both of the cancer cell growth rate and of the angiogenic phenotype, in the neoplastic cervix following inhibition of PDGF signaling, prompted us to investigate possible changes in growth and angiogenic regulatory signals. The expression of a panel of candidate genes encoding proliferative and antiapoptotic signaling molecules, based on a literature search for factors known to be of importance for cervical cancer growth, as well as prototypical angiogenic factors, was analyzed by quantitative RT-PCR in normal mouse cervix, and in CIN3 and SCC lesions, as depicted in Figure 4A. The genes coded with white bars indicate there was no change in expression during tumor progression, whereas the red bars symbolize the genes up-regulated during tumor progression, and green bars those down-regulated during tumor progression. The data are presented in more depth in Table S1. To assess changes in gene expression effected by treatment with imatinib, cervical tissues from HPV/E_2_ mice treated for 2 wk with vehicle control or with imatinib were examined. The analysis revealed that expression of fibroblast growth factor (FGF) type-7 (FGF-7; keratinocyte growth factor) was elevated throughout the progression of cervical neoplasias in HPV/E_2_ mice (Figure 4B; Student *t*-test, *t* = 15.9, *p* < 0.001), and its expression was decreased by 35% in imatinib-treated versus control tumors (Figure 4A and 4B; Student *t*-test, *t* = 10.9, *p* < 0.001); down-regulation of FGF-7 may therefore contribute to the observed antiproliferative effect of imatinib. The small mass of the mouse cervix and a lack of reagents with high sensitivity and specificity precluded biochemical quantiation of the protein levels of FGF-7 during the course of cervical tumor progression. Conspiciously, a large proportion of human cervical SCC lesions reportedly express FGF receptor-2IIIB, the receptor for FGF-7, in contrast to normal cervical epithelium [44]. Increased expression of insulin-like growth factors −1 and −2, and hepatocyte growth factor, was observed during neoplastic progression; notably, however, no substantive alterations in the expression of these factors, or of other candidate tumor growth factors and receptors, resulted from the treatment with imatinib (Figure 4A and Table S1).

A similar survey of angiogenic signaling factors revealed that VEGF-A, FGF-1, angiopoietin-1 and −2, and Ephrin A1 and B2 were similarly expressed in the cervical neoplasias of imatinib-treated versus untreated mice (Figure 4A and Table S1). Among these, only angiopoietin-1 was appreciably up-regulated during tumor progression (Figure 4A and Table S1). In contrast, FGF-2 (basic FGF) was not only appreciably up-regulated in the neoplastic cervix of HPV/E_2_ mice, compared to control N/E_2_ mice (Figure 4A and 4C; Student *t*-test, *t* = 13.7, *p* < 0.001), but its mRNA levels were reduced by 65% in CIN3 lesions from HPV/E_2_ mice treated with imatinib (Figure 4A and 4C; Student *t*-test, *t* = 18.7, *p* < 0.0001). The down-regulation of FGF-2 upon treatment with imatinib was further confirmed at the protein level by immunoprecipitation of FGF-2 from lysates of cervical tissue followed by western blotting (Figure 4D).

### FGF-2 Functionally Contributes to the Angiogenic Phenotype

We next assessed expression of the principal mitogenic signaling receptor for FGF-2, FGF Receptor-1 (FGFR1), by immunohistochemical staining. FGFR1 was found to be predominantly expressed by endothelial cells in the tissue underlying the dysplastic and invasive epithelium (Figure 4E), supporting the proposition that down-regulation of FGF-2 in response to treatment with imatinib is serving to inhibit angiogenesis driven in part by FGFR1 signaling in tumor endothelial cells.

To address the hypothesis that FGF-2 is causally involved in the angiogenic phenotype of HPV/E_2_ mice, we employed adenoviral delivery of a soluble form of an FGF receptor-2-Fc fusion protein (FGF-trap [30]). Typically, the adenovirus will be highly produced in the liver of infected mice, from where it will sustain high levels of production of the neutralizing FGF-trap into the blood stream for approximately 2 wk. HPV/E_2_ mice were humanely killed 2 wk after injection of adenovirus at the age of 4 mo. Treatment with FGF-trap produced a similar reduction in blood vessel density of cervical lesions to that resulting from treatment with imatinib (Figure 4F; Student *t*-test, *t* = 5.8, *p* < 0.001). Thus, we conclude that the down-regulation of FGF-2 expression in the cervixes of HPV/E_2_ mice is in large part responsible for the impaired angiogenesis produced by treatment with imatinib. However, we cannot exclude indirect effects on the angiogenic phenotype from inhibition of FGF-7 by FGF-trap.

### FGF-2 Is Expressed by Stromal Fibroblasts in HPV/E_2_ Mice

**Figure 5:**
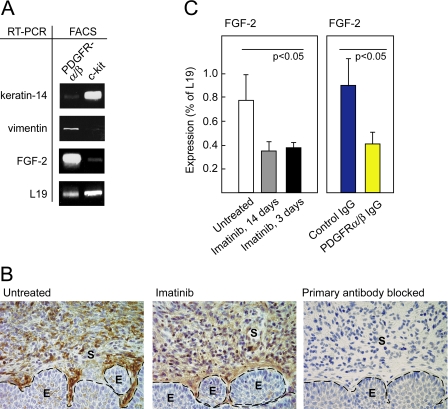
FGF-2 Is Expressed by CAFs and Repressed by Specific Inhibition of PDGF Receptor Signaling (A) Analysis of cells isolated by FACS from the cervixes of 3.5-mo-old HPV/E_2_ mice by sorting for expression of PDGFR-α and -β or c-kit. RT-PCR was performed to assess the expression of FGF-2, the squamous epithelial marker K14, the fibroblast cell marker vimentin, and the housekeeping gene *L19* as a loading control. (B) Representative immunohistochemical staining of FGF-2 in the transformation zone of the uterine cervixes of HPV/E_2_ mice that had or had not been treated with imatinib displaying CIN3 lesions. The expression pattern was analyzed in at least five different mice of similar histological stage from each treatment group. Parameters: 400× magnification; dotted line marks epithelium-stroma boundary. As a control for specificity, the primary antibody was pre-blocked by incubation with recombinant mouse FGF-2 . E, epithelium; S, stroma. (C) Expression of FGF-2 analyzed by quantitative RT-PCR following a long (14 d) and a short (3 d) treatment with imatinib (Student *t*-test, *t* = 3.5, *p* < 0.05). Expression of FGF-2 analyzed by quantitative RT-PCR following a 3-d treatment with control IgG or inhibitory antibodies against PDGFR-α and PDGFR-β (Student *t*-test, *t* = 3.1, *p* < 0.05). Note that for technical reasons, a different primer set was used in this experiment compared to the experiment shown in Figure 4C, yielding different absolute values of expression (for details, see Materials and Methods). Error bars indicate the standard error of the mean.

Having functionally implicated FGF-2 in the angiogenic phenotype, we sought to identify the cell type in cervical neoplasias and cancer that expressed FGF-2, by using FACS. Total RNA was extracted from the pools of cells labeled positively for either of the imatinib receptor tyrosine kinase targets, i.e., for PDGF receptor-α plus PDGF receptor-β, or for c-kit, and subjected to semiquantitative RT-PCR analysis to establish the identity of the cells. The pool of cells sorted for expression of c-kit was positive for expression of the basal squamous epithelial gene *K14*, but not for the mesenchymal gene *vimentin*, indicating that c-kit is expressed by a subset of cervical keratinocytes (Figure 5A). In contrast, the pool of cells sorted for expression of PDGF receptors contained the transcripts for the mesenchymal gene *vimentin*, but not *K14*, in accordance with earlier experiments showing the PDGF receptors were selectively expressed in CAFs and pericytes (Figures 1A and 5A). The transcript for FGF-2 was predominant in the pool sorted for expression of PDGF receptors, indicating that FGF-2 was largely produced by PDGF receptor-expressing cells (Figure 5A). Next, we employed immunostaining of tissue sections to visualize the cells expressing FGF-2 in the cervixes of HPV/E_2_ mice. FGF-2 was found to be predominantly expressed by stromal cells, in particular the subset of cells in proximity to the basal keratinocytes (Figure 5B). In accordance with the diminished FGF-2 mRNA levels seen following treatment with imatinib, the staining intensity for FGF-2 was reduced in tissue sections from imatinib-treated mice (Figure 5B). To ascertain which stromal cell type expressed FGF-2, we performed coimmunostaining of FGF-2 and markers for various prevalent cell types within the cervix. FGF-2 expression colocalized with expression of PDGF receptor-α and vimentin, indicating that FGF-2 is expressed by CAFs, but not with markers for endothelial cells, leukocytes, macrophages, mast cells, NK cells, or dendritic cells (Figure S4).

### PDGF Signaling Regulates FGF-2 Expression in Stromal Fibroblasts

We performed two experiments aimed to further assess the hypothesis that a signaling pathway involving PDGF receptors regulates expression of the angiogenic factor FGF-2 in stromal fibroblasts. In one approach, we treated HPV/E_2_ mice with neutralizing antibodies to both PDGF receptors [32], in a molecular efficacy (target modulation) trial. We first determined that a brief 3-d treatment with imatinib was sufficient to suppress expression of FGF-2 similarly to that of 2–6 wk of daily imatinib treatment (Figure 5C; Student *t*-test, *t* = 3.5, *p* < 0.05). Then we treated HPV/E_2_ mice for 3 d with a cocktail of two function-blocking antibodies for PDGF receptor-α and PDGF receptor-β, which reduced levels of FGF-2 mRNA in the neoplastic cervix to a level comparable to that produced by treatment with imatinib (Figure 5C; Student *t*-test, *t* = 3.1, *p* < 0.05). In contrast, treatment with control IgG for 3 d had no effect. Similarly, the expression of FGF-7 was reduced in the cervixes from mice treated with neutralizing PDGF-receptor antibodies, compared with control IgG (unpublished data). Thus, we conclude that imatinib modulates the expression of FGF-2 and −7 through inhibition of PDGF receptors. In a second approach, we stimulated cultured fibroblasts with PDGF ligands. Both PDGF-AA (a PDGF receptor-α ligand) and PDGF-BB (a PDGF receptor-β ligand) resulted in up-regulated expression of FGF-2 in fibroblasts (Figure S5).

In regard to possibly broader effects of suppressing PDGF receptor signaling in CAFs, we did not observe significant reductions in the cellularity of the neoplastic stroma, as evidenced histologically or by vimentin expression (Figure S6 and unpublished data), although there was a modestly increased incidence of apoptosis (unpublished data). Thus we infer that imatinib was primarily interfering with CAF effector functions dependent on PDGF signaling, but not with CAF viability in CIN or SCC lesions per se. This result contrasts with reports [18,19] in which CAF function and survival was reported to be PDGF-dependent in subcutaneous tumor xenotransplant models. Notably, in such cell transplant models, CAFs must be recruited from the ectopic subcutaneous microenvironment. By contrast, in many organized epithelia, including the squamous epithelia of the cervix, an abundant fibroblastic stroma is normally present. Thus, during tumorigenesis, we infer that the normal stromal fibroblasts in the cervix become activated CAFs in response to PDGF signaling, but do not strictly depend on it for survival, suggestive of significant differences in the regulation of CAFs populating distinct tumor microenvironments. Collectively, our data indicate that PDGF receptor signaling in cervical CAFs dictates the up-regulation of FGF-2 and FGF-7 during cervical carcinogenesis, such that PDGF receptor inhibitors suppress their expression with consequent functional impairment of angiogenic and neoplastic phenotypes.

### PDGFR and FGF-2 Are Similarly Up-Regulated in Human Cervical Neoplasias

**Figure 6:**
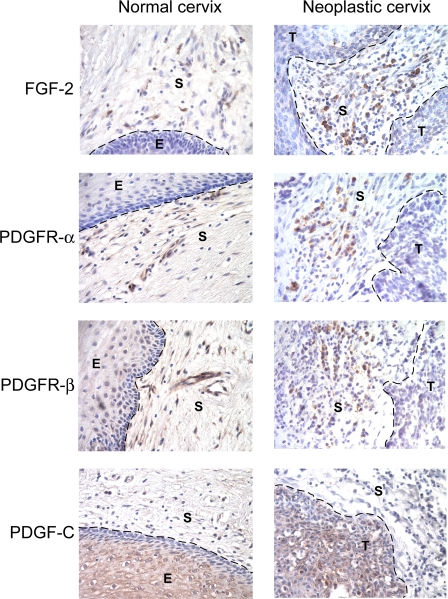
PDGF Receptors and FGF-2 Are Expressed in Human Normal Cervix and in Cervical SCC Expression of PDGF receptors and FGF-2 was assessed in human hysterectomy samples and in human cervical SCC lesions by immunohistochemistry. Representative pictures from analysis of a total of three separate human normal cervixes and SCC lesions are shown. PDGF receptor-α was exclusively expressed by stromal cells underlying the epithelium, whereas PDGF receptor-β was expressed by stromal cells and pericytes. Similarly, FGF-2 was expressed by stromal cells. Although qualitative, the staining intensity of FGF-2 is clearly lower in all of the normal human samples than in comparable analyses of neoplastic human cervixes, and consistent with the results from the mouse (Figures 4C and 5B, and unpublished data), where FGF-2 is expressed in the normal cervical stroma at low levels and clearly up-regulated in the neoplastic cervix. As in the mouse model, PDGF-CC was predominantly expressed by the cervical epithelium. Parameters: 400× magnification; dotted line marks epithelium-stroma boundary. E, epithelium; S, stroma; T, tumor.

FGF-2 and its mRNA are reportedly elevated in cervical cancers in humans [26,45,46]. Moreover, a study of human cervical carcinomas employing in situ hybridization previously reported that FGF-2 was expressed by stromal fibroblasts [47]. Extending upon these studies, we performed immunohistochemical staining of high-grade dysplastic lesions (HSIL/CIN3) or carcinomas of the human cervix, revealing prominent expression of FGF-2 in stromal cells (Figure 6; and unpublished data). Parallel analysis of the expression pattern of PDGF receptors in human cervical lesions revealed an expression pattern in the stroma similar to that of FGF-2 (Figure 6). Similar to the HPV/E_2_ mice, PDGF-CC was abundantly expressed by the neoplastic epithelium, consistent with a paracrine signaling circuit operating between the tumor cells and CAFs (Figure 6). Additionally, we analyzed normal human cervical samples derived from hysterectomies. Much as in the normal mouse cervix, the expression of the PDGF receptors was detectable in stromal fibroblasts and pericytes, and PDGF-CC was expressed by the epithelial compartment (Figure 6). Recognizing the qualitative nature of the analysis of archival human samples, the expression of both PDGF receptors and FGF-2 appears to be lower in normal versus cancerous stromal fibroblasts, consistent with the clear evidence in the mouse that PDGF signaling up-regulates FGF-2 in the neoplastic and cancerous stroma. Independent validation of our immunohistochemical analyses of human cervical cancer specimens comes from recently published data on the Web site of the Human Protein Atlas project [48] (http://www.proteinatlas.org/) (Figure S7). Notably, FGF-2, PDGF receptor-α, and PDGF receptor-β were found to be expressed in the stroma of 9/11, 12/12, and 9/9 cervical cancers, respectively, whereas expression in the overt squamous cancer cells was not consistently detected, the exception being a single case that expressed PDGF receptor-β (Figure S7 and Table S2). Collectively, these results and conclusions contradict a recent study of human cervical cancer cell lines and biopsy specimens, based solely on immunohistochemical analysis using antibodies with poorly characterized specificity. That report concluded that the PDGF receptors were expressed in a series of cultured human cervical cancer cell lines and in the tumor cell compartment of approximately half of the human cervical cancer specimens analyzed [49]. In contrast, we have been unable to detect expression of either PDGF receptor by RT-PCR or western blot analysis using validated antibodies, nor growth inhibition by imatinib, during careful analysis of three of the cervical cancer cell lines used in this study [49] (Figure S8). Moreover, and consistent with our analyses, meta-analysis of the gene expression profile of human cervical cancer cell lines in the National Center for Biotechnology Information (NCBI) GEO database revealed no detectable transcripts for either of the PDGF receptors in three separate studies of HeLa cells (unpublished data), one of the cell lines stated to express PDGFR in the report in question [49]. Our data, as well as that from the Human Protein Atlas project, from analysis of human cervical cancer cell lines and human tissues are consistent with the more definitive analysis in the mouse, indicating that PDGF receptors are predominantly expressed by stromal fibroblasts and pericytes. We leave open the possibility that certain human cervical carcinomas, for example ones that have progressed from the common squamous cell carcinoma state through an epithelial-mesenchymal transition (EMT) to a spindle cell stage, might express the PDGF receptors, a possibility that deserves future analysis with well-validated reagents. Collectively, the data suggest commonality between our genetically engineered mouse model and the prevalent form of human cervical cancer, and encourage the possibility that our findings have translational relevance to mechanisms and therapeutic interventions in human cervical neoplasia and cancer.

## Discussion

Herein we demonstrate the functional role of PDGF receptor signaling in cancer-associated fibroblasts and pericytes for cancer of the uterine cervix using a mouse model of HPV16-induced cervical carcinoma. We assessed the functional importance of PDGF receptor signaling via preclinical trials with the selective PDGF receptor inhibitor imatinib, which slowed the progression of lesions from premalignant to invasive, and impaired the growth of existing tumors. Moreover, the angiogenic phenotype of both premalignant cervical neoplasias and invasive carcinomas was affected: the treated lesions exhibited diminished blood vessel density and reduced pericyte coverage. In seeking the mechanism behind the impaired tumor growth and angiogenesis, we found that the production by CAFs of FGF-7, an epithelial cell growth factor, and of FGF-2, an angiogenic factor, was substantively diminished by imatinib. Recognizing that imatinib inhibits multiple kinases, we used neutralizing antibodies to the PDGF receptors to demonstrate that suppression of FGF-2 and FGF-7 expression was a specific consequence of PDGFR blockade. Additionally, we used an FGF ligand trap (an FGFR2-Fc fusion) to demonstrate that FGF-2 (and possibly FGF-7) signaling was indeed functionally involved in regulating angiogenesis in the neoplastic cervix, in that neovascularization was markedly reduced in mice treated with this FGF inhibitor. Notably, human cervical neoplasias and carcinomas coexpressed FGF-2 and PDGF receptors in stromal cells, revealing a close similarity between this mouse model and the human disease. We have, therefore, demonstrated the utility of pharmaceutical targeting of CAFs, using a PDGF receptor inhibitor, to impair the progression to and subsequent growth of cervical carcinomas using a genetically engineered mouse model.

**Figure 7:**
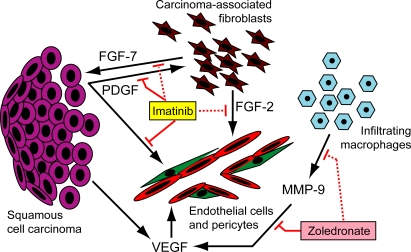
Schematic Model of the Angiogenic Circuitry Operative during Malignant Progression in the Cervical Transformation Zone of HPV/E_2_ Mice The transformed epithelial cells (purple) secrete VEGF, which becomes sequestered in the matrix until released by the action of MMP-9, produced by infiltrating macrophages (blue); the bioavailable VEGF then acts directly on endothelial cells (red) to stimulate angiogenesis. PDGF ligands are predominantly produced by the squamous epithelium and stimulate production of FGF-2 and FGF-7 by carcinoma-associated fibroblasts (brown) that express PDGF-receptors. FGF-2 stimulates angiogenesis by direct action on endothelial cells, and PDGF further promotes the angiogenic process by inducing pericyte (green) recruitment and association with newly formed blood vessels. FGF-7 may signal to the cervical carcinoma cells. Imatinib acts by inhibiting PDGF ligand-dependent PDGF receptor signaling (solid red lines), thereby repressing the production of FGF-2 and FGF-7 by carcinoma-associated fibroblasts (dotted red lines) and additionally reducing the pericyte coverage on tumor blood vessels. The clinically approved bisphosphonate zoledronate (zoledronic acid, Zometa, ZA) has been shown to suppress the expression of MMP-9 by macrophages (dotted red line), as well as to directly inhibit the proteolytic action of MMP-9 (solid red line).

PDGF ligands are expressed in a variety of tumor types, as well as by epithelial cells during embryogenesis [13,14,50]. Our study employing the PDGF receptor inhibitor imatinib indicates that PDGF plays a dual role in the angiogenic phenotype of cervical SCC. A model for the angiogenic regulatory circuits contributing to neoplastic progression and tumor growth in the cervix of HPV/E_2_ mice, as elucidated by our studies (this work, and [24]), is shown in Figure 7. Consistent with previous studies in other tumor types [35,51,52], the data suggest that PDGF helps maintain pericyte support of the tumor vasculature. In addition, we have identified PDGF receptor signaling in CAFs as a mediator of the angiogenic response in tumors, by virtue of up-regulating expression of the proangiogenic factor FGF-2. We infer that both cellular targets, CAFs and pericytes, are contributing to the angiogenic phenotype and to its impairment by imatinib. In a model of islet cell carcinogenesis, imatinib was not antiangiogenic when used as monotherapy; rather it destabilized pericyte association and rendered coadministered endothelial cell inhibitors more effective in vessels lacking pericyte coverage [35–37]. In notable contrast, imatinib is directly antiangiogenic in the cervix as monotherapy, a result we have functionally attributed to FGF signaling from PDGFR-expressing CAFs (which are rare in the islet carcinoma model). Additionally, PDGF signaling elevates expression of FGF-7, which we infer may directly stimulate the cervical cancer cells, a possibility that deserves future investigation. Our findings are in agreement with earlier studies demonstrating production of both FGF-2 and FGF-7 by CAFs in response to PDGF [6]. We do not exclude the possibility that there will prove to be additional PDGF- and/or imatinib-regulated cross-talk between CAFs, carcinoma cells, and the other cell types constituting the tumor microenvironment, potentially embellishing the critical pathway we have elucidated.

The notion of targeting CAFs as a means to interfere with the growth of tumors is attractive, since the stromal compartment is a rich contributor of various growth- and invasion-promoting activities [3,4,7]. The capability of CAFs to produce proangiogenic factors, including FGFs and VEGF, has been demonstrated in several recent studies [18,53–58]. We have now implicated FGF-2 produced by CAFs in response to PDGF signaling as a new proangiogenic regulatory axis in the neoplastic cervix. It will be of interest to assess expression of FGF-2 and its concordance with PDGF receptor expression in CAFs of other tumor types. Indeed, FGF-2 was found to be highly up-regulated and exclusively expressed by stromal fibroblasts in breast carcinomas, compared to adjacent normal tissue [59]. Despite indications of functional and molecular genetic heterogeneity in different tissues, CAFs are likely to have common determinants, including a dependence on PDGF signaling [18,19]. PDGF receptors are expressed by CAFs in a majority of tumor types (J. Paulsson, T. Sjöblom, C-H. Heldin, A. Östman et al., unpublished data; and [60–63]), and as such, our results may have broad clinical implications for targeting of stromal cells as a therapeutic strategy.

### Translational Perspective

The entry of targeted therapeutics into the clinic has been much anticipated. However, despite successful preclinical trials, the results from clinical trials with targeted agents as monotherapy have not in general produced enduring responses. Increasingly, clinical trial designs involving combinations of drugs targeting different critical components or compartments of a tumor are being discussed to enhance efficacy and to reduce the likelihood of emergence of resistance to treatment. Our findings encourage confirmatory studies in other mouse models of cervical cancer, as well as concomitant discussions of cervical cancer clinical trials involving treatment with imatinib, or other Federal Drug Administration–approved drugs incorporating inhibitory action against the PDGF receptors, e.g. the multitargeted tyrosine kinase inhibitors sunitinib, sorafenib, or dasatinib. Moreover, combinatorial treatment regimens with imatinib and standard-of-care modalities, such as topotecan chemotherapy, or as an adjuvant to radiation therapy could be considered, perhaps buoyed by further preclinical trials in mouse models such as the one employed herein. Interestingly, several phase II studies investigating the utility of sunitinib or sorafenib in combination with chemotherapy and/or radiation therapy are currently recruiting cervical cancer patients. Importantly, rationale for such trials integrating treatment with VEGF and PDGF receptor inhibitors and chemotherapy, either in concomitant treatment strategies, or in sequential “chemo-switch” regimens, has been provided by several recent studies involving preclinical trials in mouse models [36,39,64]. Additional multitargeting strategies are likely to be forthcoming from mechanism-based preclinical trials. For example, using the HPV/E_2_ transgenic mice, we have previously demonstrated functional inhibition of VEGF receptor signaling by treatment with zoledronate (Zometa) as a strategy to impair angiogenesis, and thereby tumor growth, in cervical cancer. Zoledronate acts by reducing the expression of MMP-9 by infiltrating macrophages and by inhibiting the residual proteolytic activity of MMP-9, consequently reducing the bioavailability of VEGF and impairing angiogenesis and neoplastic progression [24]. We envision that combined treatment with imatinib and zoledronate, in effect targeting two distinct angiogenic circuits within the cervical tumor (Figure 7), might further weaken the angiogenic response and limit tumor formation and growth. Indeed, we have conducted a pilot study combining the two drugs that encourages this line of reasoning and motivates further investigation (unpublished data).

The realization that imatinib, which inhibits PDGF receptor, but not VEGF receptor signaling, and yet is demonstrably antiangiogenic (via suppressing FGF-2) and antiproliferative (likely via suppressing FGF-7), might foster consideration of clinical trial designs not feasible with the aforementioned multi-targeted inhibitors. In particular, there is increasing discussion about “sequencing” of alternating or layering on drugs with different specificities, aiming to improve efficacy and reduce toxicity. We suggest that imatinib and other selective PDGF receptor inhibitors (e.g., dasatinib) might prove beneficial when combined with VEGF receptor inhibitors and/or with chemotherapy in more flexible combinations than strictly simultaneous dosing, involving evolutions of the chemo-switch regimen [36]; thus we might imagine first normalizing the tumor vasculature with VEGF pathway inhibitors [65], then applying high-dose (standard-of-care) chemotherapy, and finally introducing PDGF receptor inhibitors, alone or in combination with VEGF inhibitors or low-dose metronomic chemotherapy. Although the number of possible combinations in drugs and regimens is challenging, we speculate that preclinical trials in mouse models as illustrated by this study could help evaluate and prioritize the possibilities.

## Supporting Information

Figure S1 Systemic Treatment with Imatinib Inhibits PDGF Receptor Activation in the Neoplastic Cervix Immunoprecipitation (IP) of PDGF receptor-α from a pool of three cervical tissue lysates derived from mice treated for 2 wk with twice daily administrations of imatinib (total dose 150 mg × kg^−1^ × day^−1^). Parallel membranes were probed for the abundance of PDGF receptor-α and calnexin to demonstrate equal loading and amount of starting material.
(1.2 MB TIF)

Figure S2 Expression of the Imatinib Target c-kit and Its Ligand Is Unaltered during Cervical Carcinogenesis in the Mouse (A) Quantitative RT-PCR analysis of expression of c-kit receptor and its ligand SCF in the cervixes of estrogen-treated normal mice (N/E_2_) or HPV/E_2_ mice with CIN3 lesions (3 mo) or SCC (5 mo).
(B) Immunostaining of neoplastic cervix for c-kit (red) revealed coexpression with markers for mast cells (mast cell tryptase, green) in the stromal compartment, as well as very weak staining of a subset of epithelial cells above the layer of basal keratinocytes. Arrow points out a mast cell for comparison of expression levels. Magnification is 400×; cell nuclei/DAPI, blue; dotted line marks epithelial-stromal boundary.
E, epithelium; S, stroma.
(3.1 MB TIF)

Figure S3 The Abundance of MMP-9–Expressing Cells, or Other Constituent Cell Types of the Neoplastic Cervix, Is Not Altered by Imatinib Therapy (A) Immunostaining of cells expressing MMP-9 (green) in the cervical transformation zone of HPV/E_2_ mice. Magnification is 200×; cell nuclei/DAPI, blue; dotted line marks epithelial-stromal boundary. E, epithelium; S, stroma.
(B) Immunostaining of the neoplastic cervix using cell-type–specific markers (green) revealed no differences in abundance following treatment with imatinib. The cell-type markers were F4/80, macrophages; CD45, leukocytes; mast cell tryptase, mast cells; CD69, NK cells; and CD11c, dendritic cells) Magnification is 400×; cell nuclei/DAPI, blue. Quantifications were performed using five mice per treatment group.
(14 MB TIF)

Figure S4 FGF-2 Is Produced by CAFs Expressing PDGF Receptor-α and Vimentin Immunostaining of the stromal compartment of the neoplastic cervix for FGF-2 (red) and for cell type specific markers (green). The markers used to identify the particular cell types are PDGF receptor-α for fibroblasts, vimentin for CAFs; CD31 for endothelial cells; F4/80 for macrophages; CD45 for leukocytes; mast cell tryptase for mast cells; CD69 for NK cells; and CD11c for dendritic cells). Magnification is 400×; cell nuclei/DAPI, blue.
(21 MB TIF)

Figure S5 Induction of FGF-2 by PDGF in Cultured Fibroblasts FGF-2 transcription was assessed following stimulation of NIH-3T3 mouse fibroblasts with PDGF-AA (100 ng/ml for 6 h in 37 °C) or PDGF-BB (100 ng/ml for 6 h in 37 °C). The analysis revealed that fibroblasts up-regulate expression of FGF-2 in response to PDGF. Expression of the housekeeping gene GAPDH was used as a control.
(418 KB TIF)

Figure S6 Stromal Cell Density Is Not Altered in the Neoplastic Cervix following Treatment with Imatinib Quantification of stromal cell density in the stroma of the transformation zone of the cervixes from groups of five mice treated, or not, with imatinib for 2 wk.
(405 KB TIF)

Figure S7 PDGF Receptors and FGF-2 Are Expressed in the Stromal Cell Compartment of an Extended Set of Cervical Cancers Representative images of immunohistochemical stainings obtained from the Human Protein Atlas project (http://www.proteinatlas.org/) demonstrate stromal expression of FGF-2 and PDGF receptors.
(12 MB TIF)

Figure S8 Lack of PDGF Receptor Expression or Functionality on Human Cervical Cell Lines (A) PCR analysis of expression of PDGFR-α and PDGFR-β by human cervical cancer cell lines (SiHa, C33-A, HeLa) demonstrated that the PDGF-receptor genes were not transcribed. Normal human fibroblasts (NF) were used as a positive control.
(B) Western blot analysis of immunoprecipitated PDGFR-α [66] using cell lysates from human cervical cancer cell lines stimulated or not with PDGF-CC demonstrated that PDGFR-α was not expressed. Normal human fibroblasts (NF) were used as a control.
(C) In vitro analysis of the growth rate of the cervical cancer cell line HeLa grown in the presence or absence of the PDGF-receptor inhibitor imatinib showed that the growth rate of HeLa cells is not altered by the presence of 4.4 μM imatinib, corresponding to the peak plasma concentration of imatinib delivered to patients at the standard dose of 400 mg/day. Porcine aortic endothelial (PAE) cells transfected with the PDGFR-α were used as a positive control for the action of imatinib.
(3.0 MB TIF)

Table S1 Expression Level of Growth Factors in the Normal and Neoplastic Cervix Total RNA was extracted from the cervixes of 5-mo-old FVB/n mice treated with estrogen (N/E_2_), from 3-mo-old HPV/E_2_ mice (CIN3), or from 5-mo-old HPV/E_2_ mice (SCC). A pool consisting of five mice from each group was assessed for gene expression using quantitative RT-PCR. The data shown represent the mean from two separate experiments, and are depicted as percent expression of the reference gene L19.
(29 KB DOC)

Table S2 Expression of FGF-2 and PDGF-Receptors in Cervical Cancer Data obtained from meta-analysis of immunohistochemical stainings performed by the Human Protein Atlas project (http://www.proteinatlas.org). Images were scored for expression in the tumor stroma or neoplastic compartment following careful examination of each specimen displayed on the Web site.
(30 KB DOC)

## Abbreviations

CAF: cancer-associated fibroblast
CIN: cervical intraepithelial neoplasia
E_2_: 17β-estradiol
FACS: fluorescence activated cell sorting
FGF: fibroblast growth factor
HPV: human papilloma virus
K14: keratin 14
MMP: matrix metalloproteinase
PDGF: platelet-derived growth factor
rt: room temperature
RT-PCR: reverse-transcription polymerase chain reaction
SCC: squamous cell carcinoma
VEGF: vascular endothelial growth factor
