# Supplementary figures and images for: Functions of Paracrine PDGF Signaling in the Proangiogenic Tumor Stroma Revealed by Pharmacological Targeting
Source: PLoS Med. 2008 Jan 29;5(1):e19. doi: 10.1371/journal.pmed.0050019 (PMC2214790; doi:10.1371/journal.pmed.0050019)

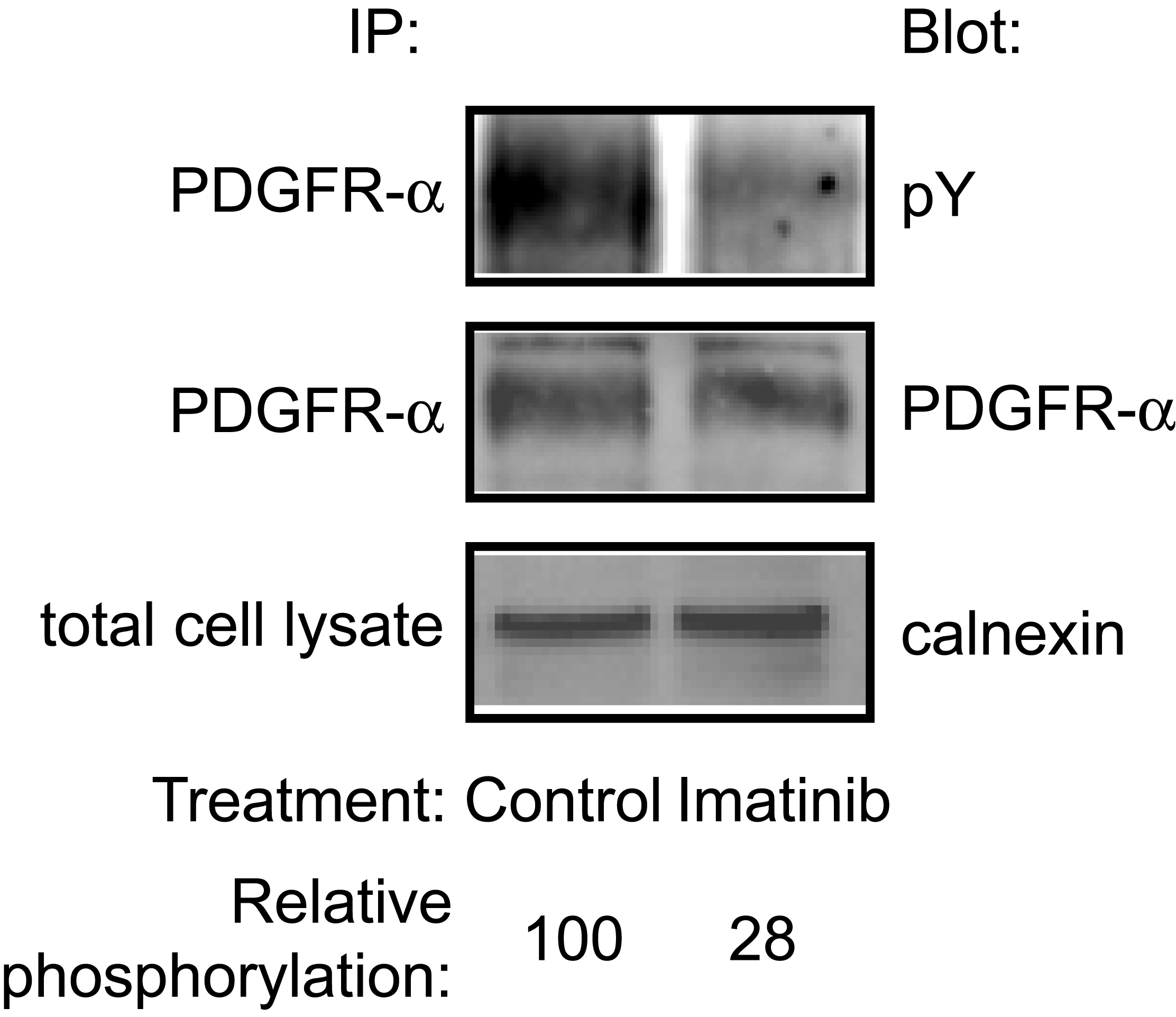

Supplement: Figure S1 — Immunoprecipitation (IP) of PDGF receptor-α from a pool of three cervical tissue lysates derived from mice treated for 2 wk with twice daily administrations of imatinib (total dose 150 mg × kg−1 × day−1). Parallel membranes were probed for the abundance of PDGF receptor-α and calnexin to demonstrate equal loading and amount of starting material. (1.2 MB TIF) [file pmed.0050019.sg001.tif]

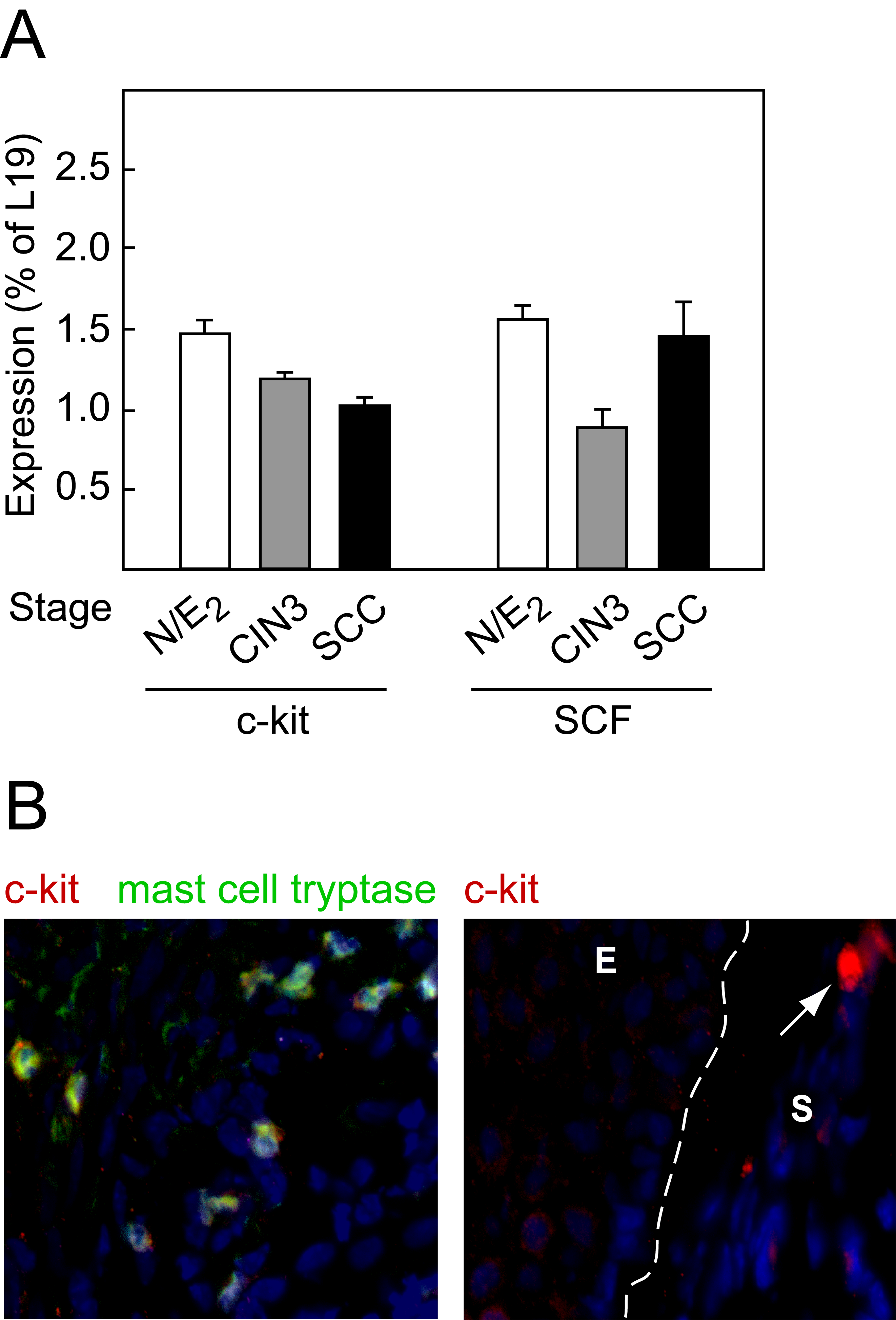

Supplement: Figure S2 — (A) Quantitative RT-PCR analysis of expression of c-kit receptor and its ligand SCF in the cervixes of estrogen-treated normal mice (N/E2) or HPV/E2 mice with CIN3 lesions (3 mo) or SCC (5 mo). (B) Immunostaining of neoplastic cervix for c-kit (red) revealed coexpression with markers for mast cells (mast cell tryptase, green) in the stromal compartment, as well as very weak staining of a subset of epithelial cells above the layer of basal keratinocytes. Arrow points out a mast cell for comparison of expression levels. Magnification is 400×; cell nuclei/DAPI, blue; dotted line marks epithelial-stromal boundary. E, epithelium; S, stroma. (3.1 MB TIF) [file pmed.0050019.sg002.tif]

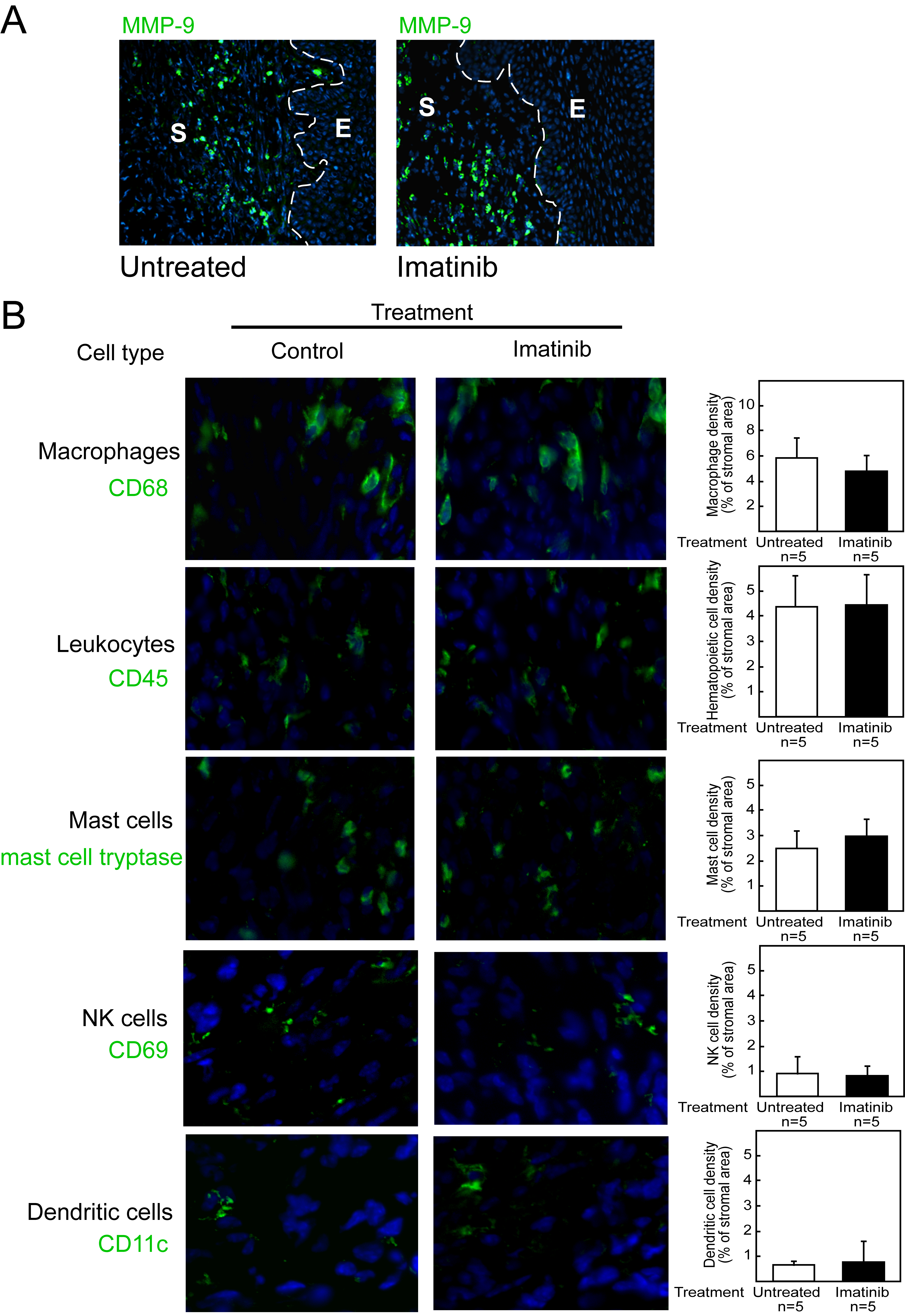

Supplement: Figure S3 — (A) Immunostaining of cells expressing MMP-9 (green) in the cervical transformation zone of HPV/E2 mice. Magnification is 200×; cell nuclei/DAPI, blue; dotted line marks epithelial-stromal boundary. E, epithelium; S, stroma. (B) Immunostaining of the neoplastic cervix using cell-type–specific markers (green) revealed no differences in abundance following treatment with imatinib. The cell-type markers were F4/80, macrophages; CD45, leukocytes; mast cell tryptase, mast cells; CD69, NK cells; and CD11c, dendritic cells) Magnification is 400×; cell nuclei/DAPI, blue. Quantifications were performed using five mice per treatment group. (14 MB TIF) [file pmed.0050019.sg003.tif]

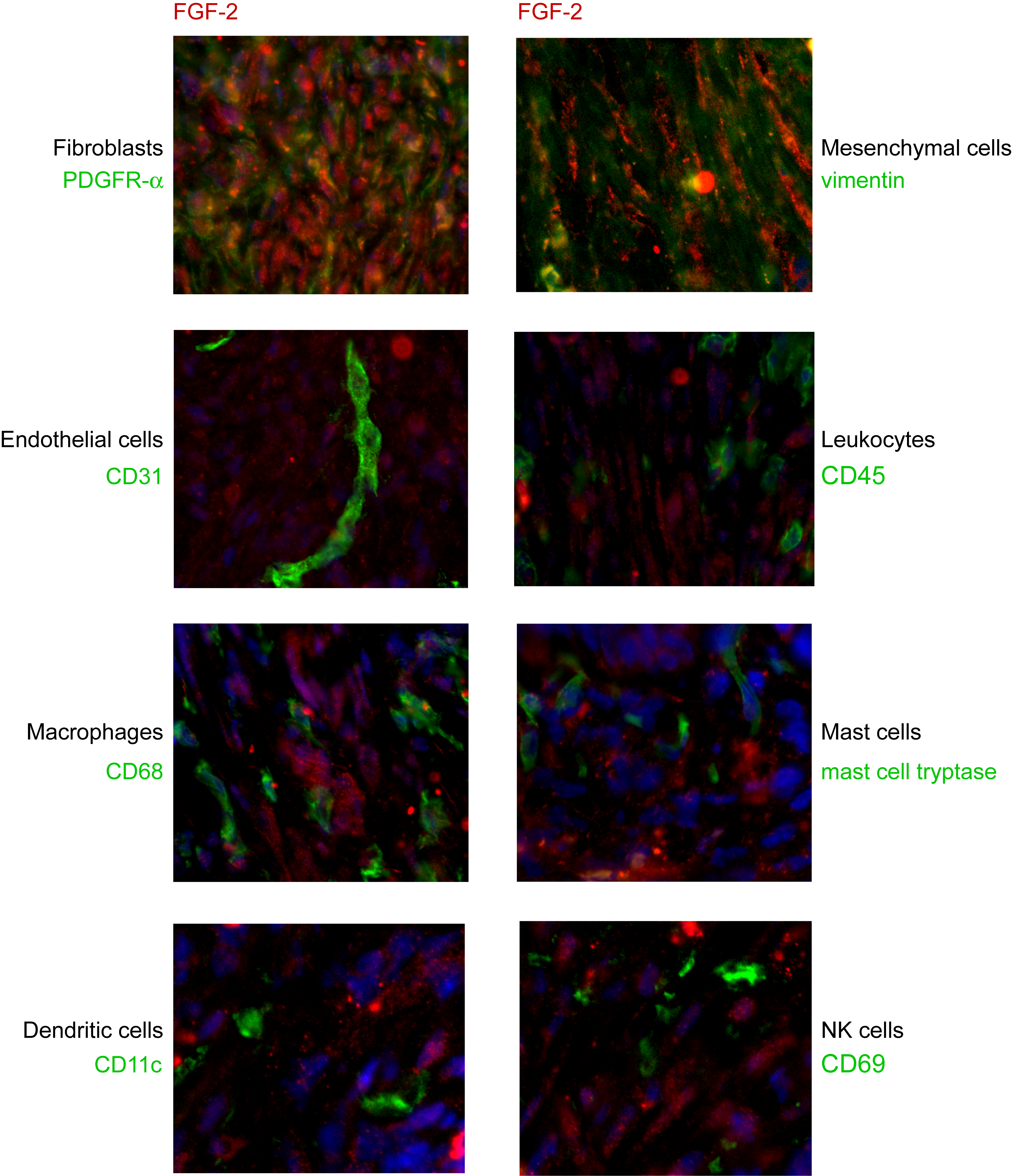

Supplement: Figure S4 — Immunostaining of the stromal compartment of the neoplastic cervix for FGF-2 (red) and for cell type specific markers (green). The markers used to identify the particular cell types are PDGF receptor-α for fibroblasts, vimentin for CAFs; CD31 for endothelial cells; F4/80 for macrophages; CD45 for leukocytes; mast cell tryptase for mast cells; CD69 for NK cells; and CD11c for dendritic cells). Magnification is 400×; cell nuclei/DAPI, blue. (21 MB TIF) [file pmed.0050019.sg004.tif]

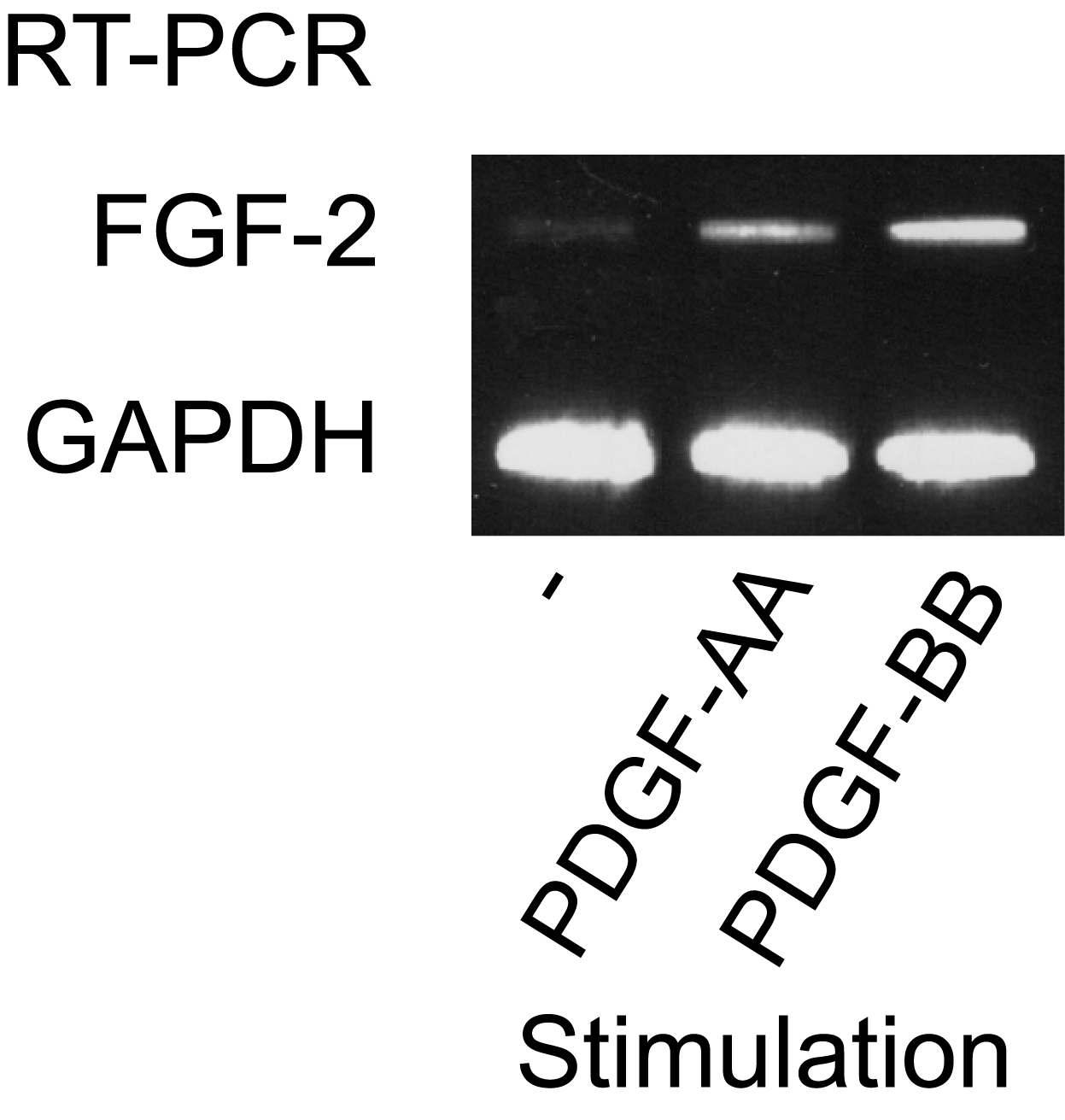

Supplement: Figure S5 — FGF-2 transcription was assessed following stimulation of NIH-3T3 mouse fibroblasts with PDGF-AA (100 ng/ml for 6 h in 37 °C) or PDGF-BB (100 ng/ml for 6 h in 37 °C). The analysis revealed that fibroblasts up-regulate expression of FGF-2 in response to PDGF. Expression of the housekeeping gene GAPDH was used as a control. (418 KB TIF) [file pmed.0050019.sg005.tif]

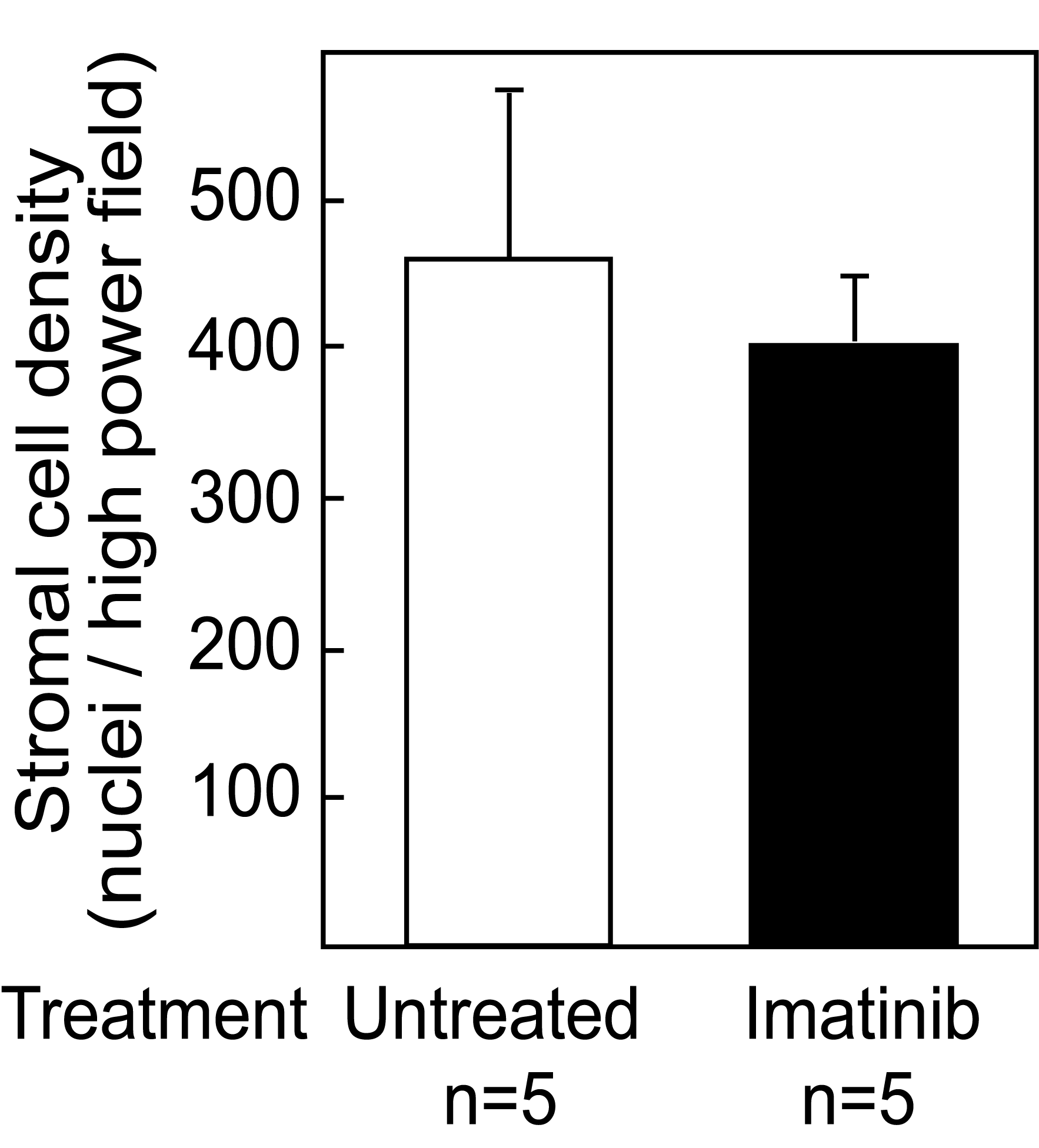

Supplement: Figure S6 — Quantification of stromal cell density in the stroma of the transformation zone of the cervixes from groups of five mice treated, or not, with imatinib for 2 wk. (405 KB TIF) [file pmed.0050019.sg006.tif]

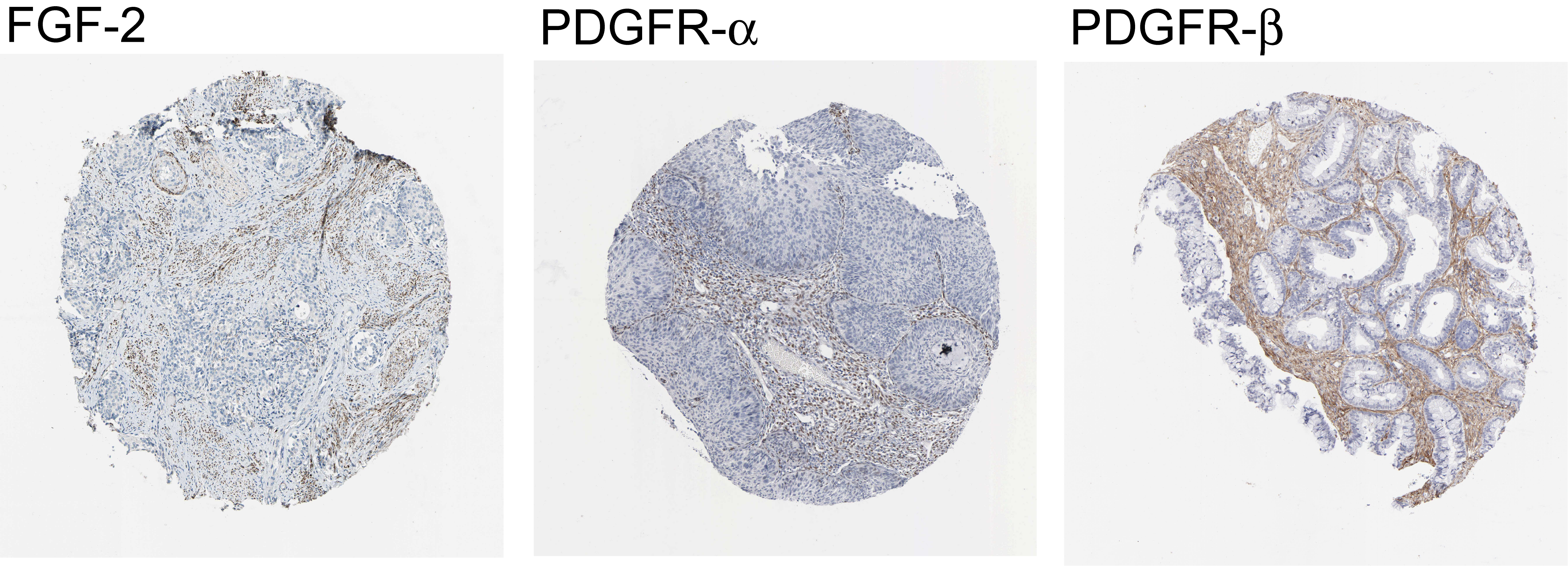

Supplement: Figure S7 — Representative images of immunohistochemical stainings obtained from the Human Protein Atlas project (http://www.proteinatlas.org/) demonstrate stromal expression of FGF-2 and PDGF receptors. (12 MB TIF) [file pmed.0050019.sg007.tif]

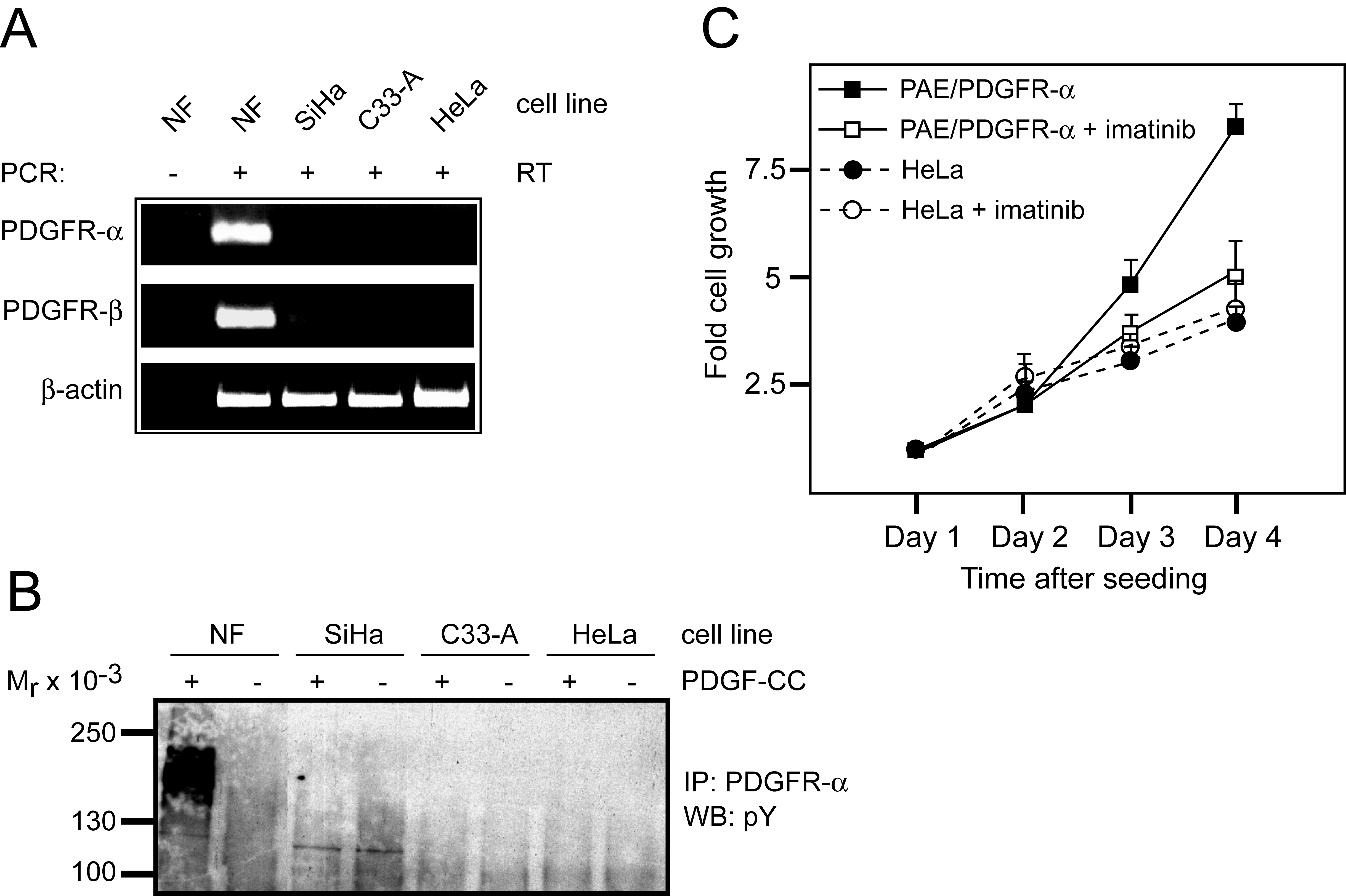

Supplement: Figure S8 — (A) PCR analysis of expression of PDGFR-α and PDGFR-β by human cervical cancer cell lines (SiHa, C33-A, HeLa) demonstrated that the PDGF-receptor genes were not transcribed. Normal human fibroblasts (NF) were used as a positive control. (B) Western blot analysis of immunoprecipitated PDGFR-α [66] using cell lysates from human cervical cancer cell lines stimulated or not with PDGF-CC demonstrated that PDGFR-α was not expressed. Normal human fibroblasts (NF) were used as a control. (C) In vitro analysis of the growth rate of the cervical cancer cell line HeLa grown in the presence or absence of the PDGF-receptor inhibitor imatinib showed that the growth rate of HeLa cells is not altered by the presence of 4.4 μM imatinib, corresponding to the peak plasma concentration of imatinib delivered to patients at the standard dose of 400 mg/day. Porcine aortic endothelial (PAE) cells transfected with the PDGFR-α were used as a positive control for the action of imatinib. (3.0 MB TIF) [file pmed.0050019.sg008.tif]
