# Supplementary material for: Functions of Paracrine PDGF Signaling in the Proangiogenic Tumor Stroma Revealed by Pharmacological Targeting
Source: PLoS Med. 2008 Jan 29;5(1):e19. doi: 10.1371/journal.pmed.0050019 (PMC2214790; doi:10.1371/journal.pmed.0050019)
Supplement: Table S1 — Total RNA was extracted from the cervixes of 5-mo-old FVB/n mice treated with estrogen (N/E2), from 3-mo-old HPV/E2 mice (CIN3), or from 5-mo-old HPV/E2 mice (SCC). A pool consisting of five mice from each group was assessed for gene expression using quantitative RT-PCR. The data shown represent the mean from two separate experiments, and are depicted as percent expression of the reference gene L19. (29 KB DOC) [file pmed.0050019.st001.doc]

|  | **EGF** | **TGFα** | **IGF1** | **IGF2** | **HGF** | **SCF** | **VEGF-A** | **FGF1** | **Ang1** | **Ang2** | **EphnA1** | **EphnB2** |
| --- | --- | --- | --- | --- | --- | --- | --- | --- | --- | --- | --- | --- |
| **N/E_2_** | 0.28  ±0.10 | 4.51  ±0.30 | 2.15  ±0.27 | 0.01  ±0.01 | 0.11  ±0.05 | 1.47  ±0.18 | 3.86  ±0.35 | 0.36  ±0.04 | 0.05  ±0.06 | 0.24  ±0.13 | 7.91  ±1.22 | 20.9  ±1.98 |
| **CIN3** | 0.15  ±0.04 | 1.41  ±0.04 | 15.7  ±0.84 | 0.04  ±0.01 | 0.34  ±0.12 | 1.17  ±0.18 | 2.75  ±0.42 | 0.06  ±0.06 | 0.11  ±0.02 | 0.17  ±0.08 | 9.31  ±1.26 | 18.1  ±2.80 |
| **SCC** | 0.06  ±0.07 | 1.78  ±0.12 | 19.8  ±0.62 | 0.03  ±0.01 | 0.37  ±0.13 | 1.03  ±0.16 | 1.88  ±0.39 | 0.10  ±0.02 | 0.08  ±0.04 | 0.20  ±0.05 | 8.60  ±1.35 | 15.8  ±1.17 |
