# Supplementary material for: Functions of Paracrine PDGF Signaling in the Proangiogenic Tumor Stroma Revealed by Pharmacological Targeting
Source: PLoS Med. 2008 Jan 29;5(1):e19. doi: 10.1371/journal.pmed.0050019 (PMC2214790; doi:10.1371/journal.pmed.0050019)
Supplement: Table S2 — Data obtained from meta-analysis of immunohistochemical stainings performed by the Human Protein Atlas project (http://www.proteinatlas.org). Images were scored for expression in the tumor stroma or neoplastic compartment following careful examination of each specimen displayed on the Web site. (30 KB DOC) [file pmed.0050019.st002.doc]

| **FGF-2** | 9/12 | positive stroma |
| --- | --- | --- |
|  | 2/12 | negative stroma |
|  | 1/12 | undetermined stroma |
|  | 0/12 | positive tumor cells |
|  |  |  |
| **PDGFR-α** | 9/12 | positive throughout stroma |
|  | 3/12 | positive in scattered cells in stroma |
|  | 0/12 | positive tumor cells |
|  |  |  |
| **PDGFR-β** | 9/9 | positive stroma |
|  | 1/9 | positive tumor cells |
